# Faroese whole genomes provide insight into ancestry and recent selection

**DOI:** 10.7554/eLife.107428

**Published:** 2026-06-16

**Authors:** Iman Hamid, Ólavur Mortensen, Alba Refoyo-Martínez, Leivur N Lydersen, Anne-Katrin Emde, Melissa Hendershott, Katrin D Apol, Guðrið Andorsdóttir, Jonas Meisner, Kaja A Wasik, Fernando Racimo, Stephane E Castel, Noomi O Gregersen

**Affiliations:** 1 Variant Bio Inc. Seattle United States; 2 FarGen, Department of Research, National Hospital of the Faroe Islands Tórshavn Faroe Islands; 3 Centre of Health Science, University of the Faroe Islands Tórshavn Faroe Islands; 4 Section for Molecular Ecology and Evolution, Globe Institute, University of Copenhagen Copenhagen Denmark; 5 Mental Health Centre Copenhagen, Copenhagen University Hospital Copenhagen Denmark; 6 Novo Nordisk Foundation Center for Basic Metabolic Research, University of Copenhagen Copenhagen Denmark; Max Planck Institute for Biology Tübingen Germany; Max Planck Institute for Biology Tübingen Germany

**Keywords:** population genomics, positive selection, ancient admixture, runs of homozygosity, founder population, genetic architecture, Human

## Abstract

The Faroe Islands are home to descendants of a North Atlantic founder population with a unique history shaped by both migration and periods of relative isolation. Here, we investigate the genetic diversity, population structure, and demographic history of the islands by analyzing whole genome sequencing data from 40 participants in the Faroe Genome Project. This represents the first whole genome sequencing panel of this size from the Faroe Islands. We observed numerous putatively functional private alleles, including stop gain variants and high impact missense variants in the cohort. Faroese individuals had a higher proportion of their genomes contained in long runs of homozygosity than other European groups, including Finnish, suggesting a more recent or stronger bottleneck in the Faroese population. Signals of positive selection were identified at loci containing genes that play roles in vitamin D and dietary fat absorption and DNA repair, while increased diversity on lactase persistence haplotypes was observed. Fine-scale analysis of haplotype structure in present-day and ancient European genomes revealed genetic affinities with ancient Iron Age individuals from the North and West of Europe, providing evidence for potential contributions to the Faroese gene pool from Celtic and Viking populations as well as information about the temporal order in which these events happened. This study highlights the impact of evolutionary processes, such as ancient admixture, founder events, and positive selection, on the present-day genetic architecture of North Atlantic founder populations like the Faroe Islands.

## eLife digest

Our DNA contains a wealth of information about our ancestors. By examining the DNA of present-day populations, researchers can infer aspects of their history, including migration patterns, interactions with other groups, changes in population size, and adaptation to local environments.

When a small group of “founders” settles a new location, they carry only a subset of the larger population’s genetic diversity. As a result, their descendants often exhibit reduced genetic diversity and elevated frequencies of certain genetic variants, some of which may influence health, disease, or adaptation.

The Faroe Islands, a remote North Atlantic archipelago, were settled around the 9th century CE, primarily by people from Scandinavia and the British Isles. Today’s population of approximately 54,000 Faroese descends from this relatively small founding group. This distinctive demographic history – characterized by mixed ancestry, geographic isolation, and a limited number of founders – is likely reflected in the genomes of present-day Faroese individuals.

To investigate the genetic diversity, ancestry, and evolutionary history of the Faroese population, Hamid et al. conducted whole-genome sequencing on 40 individuals selected to represent the geographic diversity of the islands. Previous studies of the Faroese population were limited either to small sample sizes (fewer than 10 individuals) or to specific regions of the genome, leaving substantial gaps in our understanding of the population’s full genetic landscape. The Faroe Islands also have elevated rates of several diseases, including inflammatory bowel disease and type 2 diabetes, compared with other European populations. Understanding the unique genetic architecture and demographic history of the Faroese population may therefore provide important insight into the genetic basis of these health disparities.

The analyses revealed that Faroese individuals carry numerous rare and potentially disease-associated genetic variants that are absent from mainland European populations, including variants predicted to disrupt gene function. Compared with other European groups –including Finns, another well-known founder population – Faroese genomes contain longer runs of homozygosity (long, identical DNA segments), reflecting extensive segments of identical DNA inherited from common ancestors. This pattern suggests a stronger or more recent population bottleneck in the Faroese population.

Hamid et al. also identified signatures of natural selection in genes involved in vitamin D metabolism and dietary fat absorption (*SLC10A1*), as well as DNA repair (*POLQ*), which may reflect adaptation to the Faroese environment and traditional diet. Comparisons with ancient European genomes indicated that the Faroese derive approximately equal ancestry from Iron Age “West European” (Celtic-related) and “North European” (Viking-related) populations. Importantly, the data suggest that this genetic mixing likely occurred before the settlement of the islands rather than afterwards.

These findings have direct relevance for the Faroese population because they identify genetic variants enriched in the islands that may contribute to conditions such as glycogen storage disease, ankylosing spondylitis, and other disorders. In the future, this knowledge could help inform disease prevention strategies and clinical care in the Faroe Islands. However, the study of Hamid et al. represents only a pilot phase. A greater ongoing effort, the FarGen project, aims to integrate genomic data with detailed health records to better understand the relationship between genetics and disease in the Faroese population. More broadly, the Faroe Islands provide an important model for studying how historical isolation and founder effects shape genetic diversity and disease risk in human populations worldwide.

## Introduction

The Faroe Islands, nestled in the North Atlantic Ocean between Iceland and Norway, are home to the descendants of a North Atlantic founder population with a rich cultural heritage and a unique history shaped by both migration and periods of relative isolation. The exact settlement history of the islands is unclear, though historical records and analysis of Y-chromosome microsatellite markers point to a few founders most likely having arrived primarily from Scandinavia and the British Isles starting around the ninth century CE (Johnston, 1975; Jorgensen et al., 2004; Young, 1979). However, archeological evidence supports a possible earlier settlement of the island around the fourth to sixth centuries CE (Church et al., 2013). Studies of mtDNA reveal an excess of maternal ancestry from the British Isles, while Y chromosome studies reveal an excess of paternal ancestry from Scandinavia, suggesting sex-biased admixture between these ancestral groups during the founding of the population (Jorgensen et al., 2004; Als et al., 2006). Since settlement and early waves of migration, the Faroe Islands have been mostly isolated and have experienced minimal immigration and population growth until recent years, with a census size of about 4000 people in the 1700s (West, 1972) increasing to over 54,000 as of August 2023 (https://hagstova.fo/en/population/population/population). Patterns of genetic diversity and linkage disequilibrium from a few genetic markers suggest a founder event, followed by a historically small population size and subsequent rapid expansion (Jorgensen et al., 2002).

Overall, despite the small size and remote location of the Faroe Islands, the above evidence suggests that the genetic makeup of Faroese people may have been influenced by waves of early migration and admixture from various northwest European and Scandinavian populations. However, no population genomic studies have yet been carried out on whole–genome sequencing data to date, with the exception of one study investigating relatedness and autozygosity for a limited sample size of eight individuals (Gislason, 2023). An in-depth analysis of the genomic architecture of the Faroese may reveal how the islands’ demographic history has contributed to present-day health and disease in this population. This is of particular interest as the Faroese have a high burden of certain diseases relative to global and other European populations, such as inflammatory bowel disease and type 2 diabetes, among others (Leblond et al., 2019; Rasmussen et al., 2014; Passa, 2002; Veyhe et al., 2018; Burisch et al., 2014; Dean et al., 2014; Schwartz et al., 1995; Joensen, 2011; Hammer et al., 2016; Gregersen et al., 2016). The Faroe Genome (FarGen) Project set out to understand how the genetic diversity of the Faroese contributes to health (Gregersen et al., 2021).

Moreover, studying the genetic diversity of the Faroese provides potential insight into human migration, adaptation, and population structure in the North Atlantic region. Recently developed haplotype-based methods can provide fine resolution for inferring shared ancestry among individuals and the detection of population-specific haplotypes, but they require panels of whole-genome sequencing data (Meisner and Albrechtsen, 2022). These methods serve to better account for recent patterns of population structure and cryptic relatedness in population-based genetic studies, particularly in populations like the Faroe Islands with strong founder effects.

In this study, we present the first whole genomes sequenced as part of the FarGen Project. A set of 40 individuals were selected to optimally represent the genetic diversity of the broader Faroese population, with the aid of genealogical data reaching back to approximately 1650 CE. Whole genome sequencing (WGS) data was generated and used to identify putatively functional alleles enriched in the Faroese population, assess ancestry patterns within contemporary genomes, map signals of recent positive selection, and analyze local ancestry in a combined dataset of ancient and contemporary genomes of European descent spanning a period of 3000 years.

This study provides insight into the genetic variation, demographic history, and selection landscape of the Faroese population. These first whole genomes from FarGen may serve as a useful reference for studies on the broad implications of various evolutionary genetic processes, including bottlenecks, ancient admixture, and positive selection. More in-depth studies may expand this current study by further investigating the genetic architecture of the Faroese to unravel the demographic and evolutionary history of the population and its impact on complex traits and diseases in the islands.

## Results

### Whole genome sequencing of Faroese individuals

**Figure 1.**
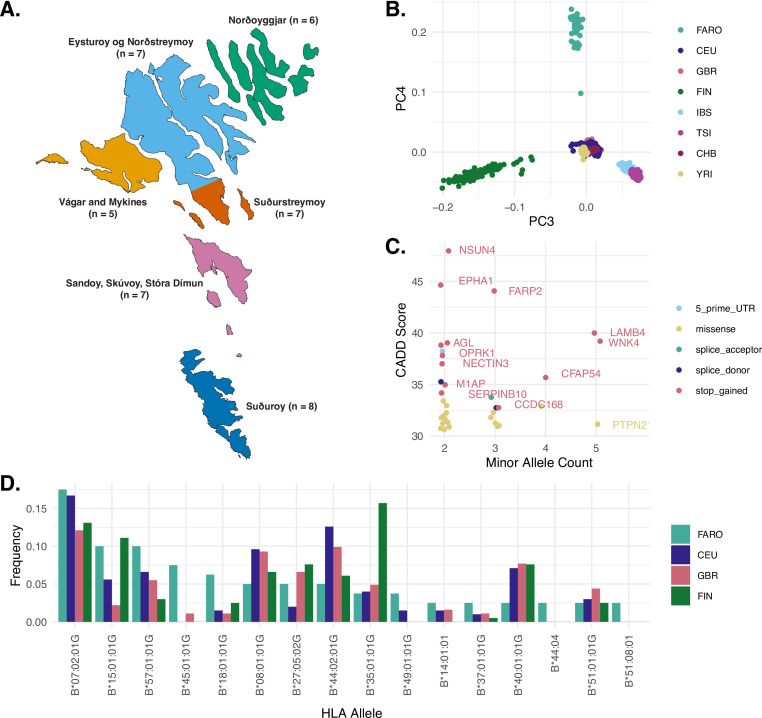
A Faroese whole genome reference. (**A**) Map of the Faroe Islands, colored by the six sampling regions. The number of minimally related FarGen participants from each region selected for whole genome sequencing is indicated. (**B**) Principal component analysis (PCA) of Faroese genomes jointly called with relevant 1000 Genomes reference data shows separation of European groups by PCs 3 and 4 (FARO, Faroese, CEU, Central Europeans, GBR, British, FIN, Finnish, IBS, Iberian, TSI, Tuscan, CHB, Han Chinese, YRI, Yoruban). (**C**) Faroese enriched putatively functional alleles visualized by minor allele count, CADD score, and Variant Effect Predictor (VEP) consequence. Variants shown are those with CADD >30 and at least two minor alleles observed in Faroese individuals, and no minor alleles observed in Finnish or Northern European reference individuals. (**D**) HLA-B allele frequencies for alleles detected at least twice in Faroese individuals. In this cohort, one minor allele corresponds to an allele frequency of 1.25%.

The Faroese Multi-Generation Register (https://www.fargen.fo/research/multi-generation-registry) was used to reconstruct a single connected genealogical tree for the 1541 participants in the first phase of the FarGen cohort (Gregersen et al., 2021; Apol et al., 2022). Individuals with fewer than six direct ancestors (two parents and four grandparents) recorded in the registry were excluded. Pairwise kinship coefficients were estimated from the genealogical tree and used to perform relatedness pruning with a threshold of 2^−6^ resulting in 332 minimally related individuals. We defined six geographical regions of the Faroe Islands using language dialects, and assigned individuals to these regions based on their place of birth (Figure 1A). A total of 40 minimally related individuals were selected for inclusion in the study, with five to eight individuals sampled from each region.

**Figure 1—figure supplement 1.**
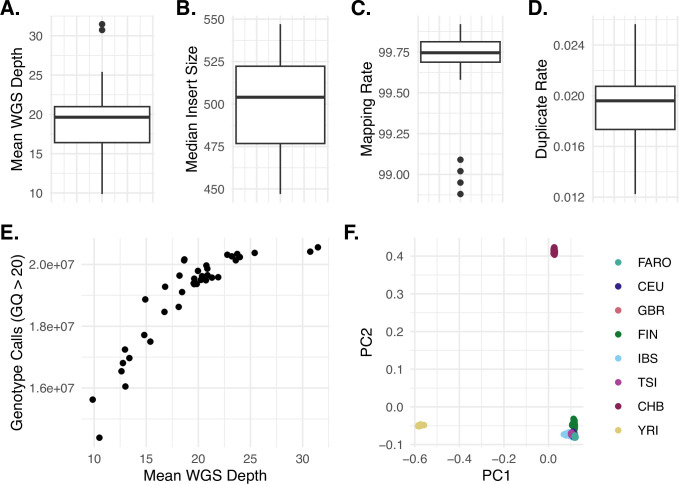
Quality control of whole genome sequencing data. (**A–D**) Distributions of QC metrics across Faroese samples sequenced (mean depth, median insert size, mapping rate, duplicate rate). (**E**) Mean whole genome sequencing (WGS) depth versus number of genotype calls with GQ >20. Singletons and calls with GT ≤20 were discarded before imputation. (**F**) Principal component analysis (PCA) of Faroese genomes jointly called with relevant 10000 Genomes reference data captures African ancestry in the first component and East Asian ancestry in the second component (FARO, Faroese, CEU, Central Europeans, GBR, British, FIN, Finnish, IBS, Iberian, TSI, Tuscan, CHB, Han Chinese, YRI, Yoruban).

The genomes of the 40 individuals were sequenced to a median depth of 20 x in the Faroe Islands at the FarGen laboratory, and they all passed quality control metrics (Figure 1—figure supplement 1, Supplementary file 1). Variant calling was performed jointly with relevant reference genome panels, including 1000 Genomes high-coverage data from Northern European (CEU & GBR, N=190), Southern European (TSI & IBS, N=214), Finnish (FIN, N=99), East Asian (CHB, N=103), and West African (YRI, N=108) individuals, and imputation was performed within the cohort using an approach we have previously described (Emde et al., 2021; Auton et al., 2015). The first component of principal component analysis (PCA) on the jointly called genotype data captured the cline between West African (YRI) and all other individuals, and the second component captured the cline between East Asian (CHB) and all other individuals (Figure 1—figure supplement 1). Principal components three and four separated European individuals, with Faroese individuals forming a distinct cluster from Finnish, Northern European, and Southern European reference groups (Figure 1B).

Variant calls were annotated with predicted functional impact and allele frequency across the reference groups. Using these annotations, we identified 35 putatively functional alleles present in our Faroese panel that are unobserved in the European mainland reference panels included in this study (CADD >30 and at least two minor alleles observed in Faroese individuals, and no minor alleles observed in Finnish or Northern European reference individuals, Supplementary file 2). These included 13 stop gain variants and 18 missense variants, and a maximum minor allele count of 5, corresponding to a frequency of 6.25% in the cohort (Figure 1C).

### HLA-B allele frequencies

Observational evidence from the FarGen project recruitment data suggests that ankylosing spondylitis (AS) may be at a higher prevalence in the Faroe Islands; however, more formal epidemiological studies are required to confirm this observation. The major histocompatibility complex (MHC) plays a role in various autoimmune diseases that may be at higher prevalence in the Faroes, including ankylosing spondylitis and other more common diseases like inflammatory bowel disease (Goyette et al., 2015; Braun and Sieper, 2023). In particular, HLA-B*27 is associated with ankylosing spondylitis (AS), with approximately 80–90% of AS patients carrying the HLA-B*27 allele (Braun and Sieper, 2023). It explains about 30% of the heritability, and ~6–8% of European populations are carriers of HLA-B*27 (Braun and Sieper, 2023; Bowness, 2015; Hanson and Brown, 2017). Using the WGS data, we genotyped human leukocyte antigen (HLA) alleles with HLA*LA (Dilthey et al., 2019). We provide HLA-B allele counts and allele frequencies in the Faroese cohort as well as the allele frequencies in 1000 Genomes British (GBR), Central European (CEU), and Finnish (FIN) individuals (Supplementary file 3). To the best of our knowledge, there have not been any larger studies of HLA-B allele frequencies in Faroese individuals, and none are currently recorded in the Allele Frequency Net Database (Gonzalez-Galarza et al., 2020).

The most frequent HLA-B allele in the Faroese cohort is HLA-B*07:02 (17.5% of haplotypes), which is a common haplotype in European populations (Figure 1D; Hurley et al., 2020; Robinson et al., 2020). We did not observe a substantial difference in HLA-B*27 allele frequency in Faroese individuals (6.25% with four B*27:05 and one B*27:01 calls) as compared to other European reference groups (2–7.6%). While 80–90% of people of European ancestry with AS carry the HLA-B*27 allele, only ~6% of HLA-B*27 carriers in the US and Europe have AS (Dillon and Hirsch, 2011; Cortes et al., 2015; Brown et al., 1996). The low frequency of the HLA-B*27 allele in this Faroese cohort in the population broadly suggests that if AS is at a higher prevalence, there may be other underlying genetic or environmental factors that explain some of the increased risk.

### Population structure and relatedness

Pairwise kinship was calculated in the cohort using *popkin* (Ochoa and Storey, 2021; Ochoa and Storey, 2019a; Ochoa and Storey, 2019b). Clustering by geography was observed when including global reference populations in the kinship calculation. The Faroe Islands have high pairwise kinship within the cohort when compared to other global populations, which may be indicative of recent bottlenecks (Figure 2—figure supplement 1A). The Faroese individuals do not show obvious clustering by region, though this is expected given the relatedness pruning during sample selection (Figure 2—figure supplement 1B).

**Figure 2.**
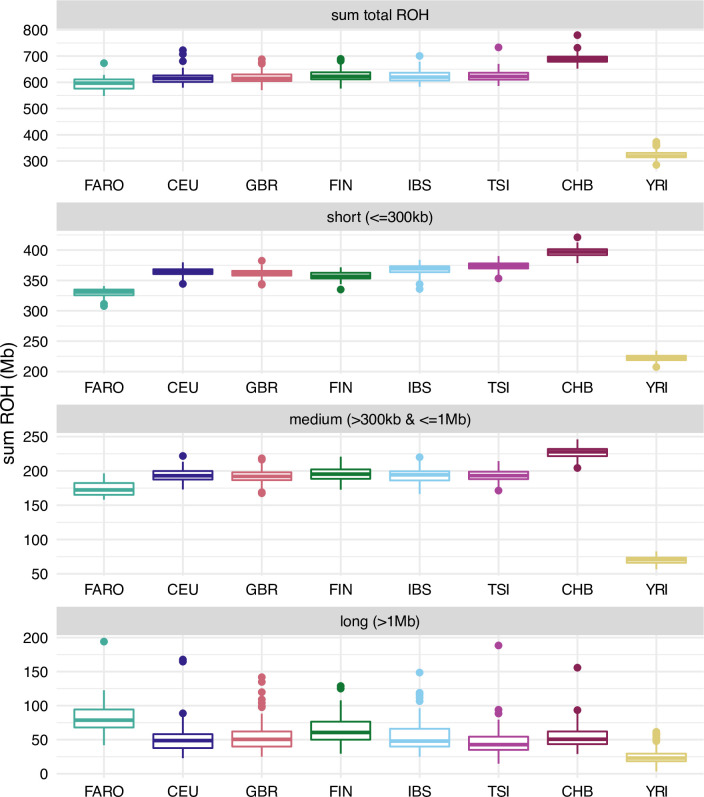
Runs of homozygosity by group. Amount of the genome (Mb) contained in runs of homozygosity (ROH) stratified by group. Top panel is the sum total of the genome contained within ROH, with the other panels showing this split by length (short, medium, and long).

We also looked at runs of homozygosity (ROHs) in the Faroese and reference cohorts (Figure 2), which can provide further insights into the demographic history of the population. As the Faroese population likely experienced a founder event during the settlement of the islands followed by rapid population size expansion in recent generations, we would expect to see more of the genome contained in ROHs compared to other global populations that have not experienced as strong a bottleneck (Ceballos et al., 2018). When looking at the sum total amount of the genome in ROHs, we found overall elevated levels of ROH in the European and Asian groups included in this analysis, most likely reflecting ancestral out-of-Africa bottlenecks for Eurasian populations (Figure 2, top panel). However, the Faroese population did not have an elevated total amount of the genome contained in ROHs compared to other European groups.

**Figure 2—figure supplement 1.**
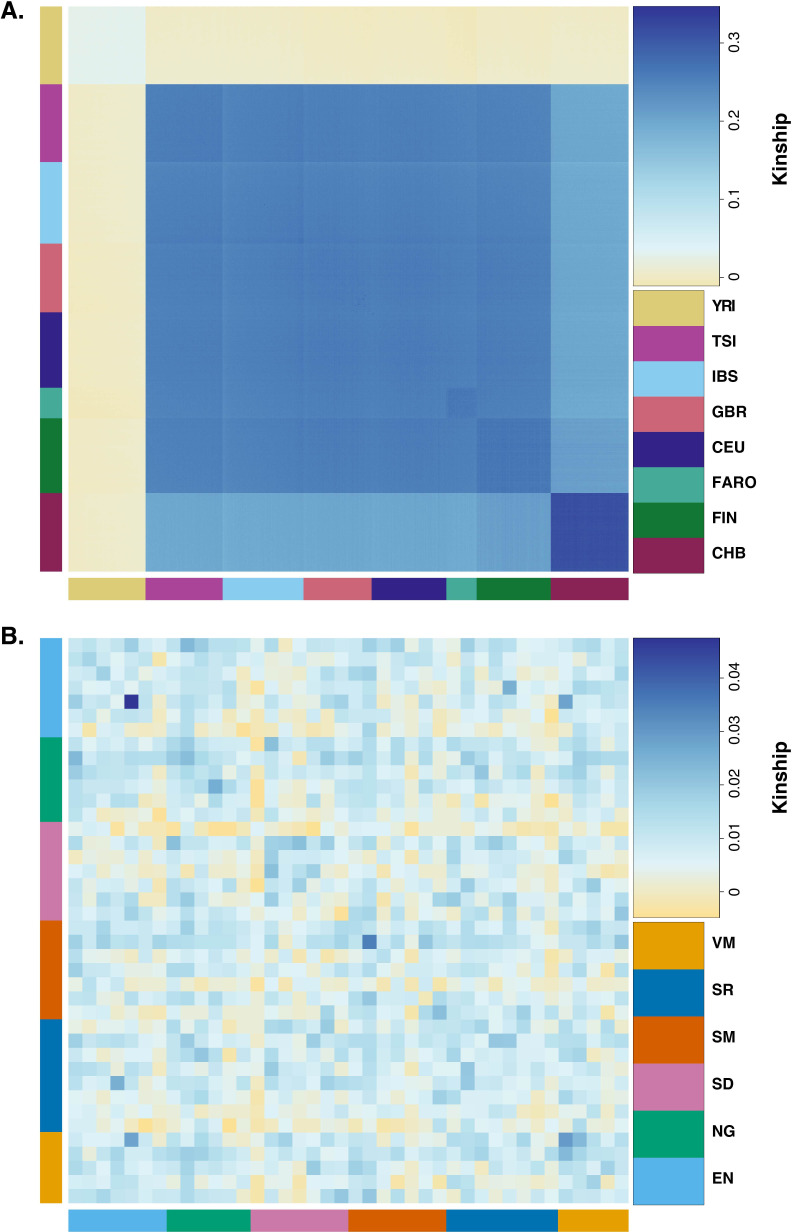
Kinship matrices between modern populations. (**A**) Kinship estimated by *popkin*, including global reference populations from the 1000 Genomes and the Faroese whole genome sequencing (WGS) cohort (Faroese, FARO). (**B**) Kinship estimated by *popkin* for the Faroese WGS cohort only for better visualization. The matrix is rescaled after subsetting the individuals, so although the scales are different, the relative kinship within the cohort remains the same for the two plots. The matrices are symmetrical and ordered by population or region label as indicated by the colored bars along the rows and columns. The diagonal of each matrix is the estimated inbreeding coefficient. The Faroes region labels are: VM = Vágar and Mykines; SR = Suðuroy; SM = Suðurstreymoy; SD = Sandoy, Skúvoy, Stóra Dímun; NG = Norðoyggjar; and EN = Eysturoy og Norðstreymoy.

**Figure 2—figure supplement 2.**
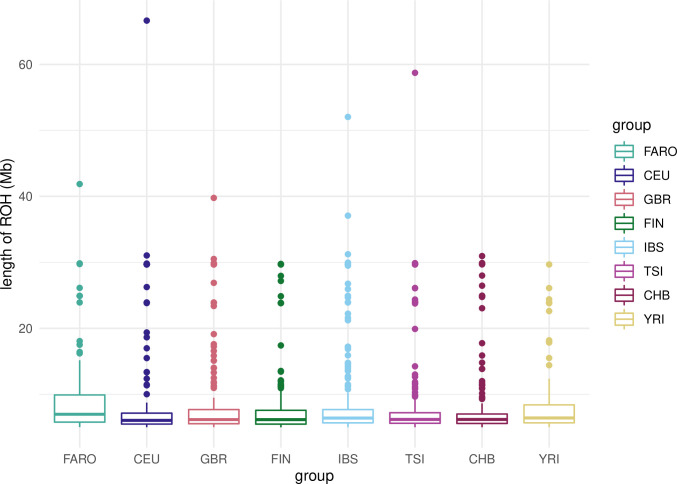
ROH length distributions by group. Length (Mb) of all runs of homozygosity (ROH) stratified by group.

To explore this further, we calculated the sum total amounts of ROH at different size categories (short, medium, long) (Figure 2, bottom three panels). We see that, on average, the Faroese individuals have less of the genome contained in short (<300 kb) and medium (>300 kb and ≤1 Mb) ROHs compared to other European groups, but more of the genome contained in long (>1 Mb) ROHs. Short and medium ROH are chunks inherited from older ancestors and reflect older events, for example, an ancient population bottleneck or founder event that has resulted in lower overall haplotype diversity, yet with enough time for recombination to break up haplotypes, while long ROH reflects chunks inherited from recent ancestors and can reflect more recent bottleneck events (Ceballos et al., 2018). Interestingly, the average amount of an individual’s genome that is contained in ROHs extending over 1 Mb in length is higher in the Faroese population (~82.5 Mb) than in the Finnish reference individuals (~63.9 Mb) and any of the other groups analyzed. Additionally, distribution in ROH lengths across all individuals stratified by group shows that, on average, there is a higher proportion of long ROH particularly in the 5–15 Mb range in the Faroese cohort relative to the other cohorts (Figure 2—figure supplement 2). This is consistent with a more recent or stronger bottleneck or founder event.

### Signals of positive selection

**Figure 3.**
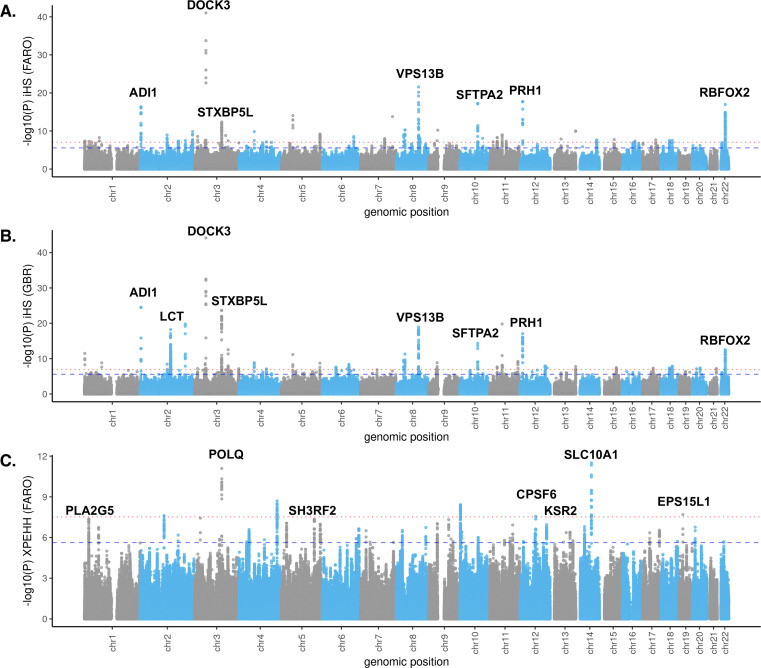
Selection scan results for Faroese and British cohorts. (**A**) Log-transformed two-tailed p-value of the standardized integrated haplotype score (iHS) in the 40 Faroese genomes (FARO). (**B**) Log-transformed two-tailed p-value of the standardized iHS for 90 British whole genome sequencing (WGS) samples from 1000 Genomes (British, GBR). (**C**) log-transformed two-tailed p-value for the standardized cross-population expected haplotype homozygosity (XPEHH) for FARO vs GBR (only positive values, which indicate selection in FARO, are plotted). Some genes in the top loci are indicated on each plot. The p-value cutoffs which correspond to a False Discovery Rate (FDR) at 0.01 and 0.001 are, respectively, indicated by the red dotted line and blue dashed line in each plot. (**A**) For iHS in FARO, these cutoffs are *p*=2.72 × 10^–6^ (FDR = 0.01) and *p*=9.20 × 10^–8^ (FDR = 0.001). (**B**) For iHS in GBR, the cutoffs are *p*=2.78 × 10^–6^ (FDR = 0.01) and *p*=1.75 × 10^–7^ (FDR = 0.001). (**C**) For XPEHH in FARO vs GBR, the cutoffs are *p*=2.35 × 10^–6^ (FDR = 0.01) and *p*=3.01 × 10^–8^ (FDR = 0.001). See Methods for details on p-value and FDR estimation.

We investigated signals of recent or ongoing positive selection in this Faroese cohort by calculating both the integrated haplotype score (iHS) (Voight et al., 2006) and cross-population expected haplotype homozygosity (XPEHH) using *hapbin* (Maclean et al., 2015). The sample size of the WGS cohort is relatively small (n=40), so our ability to detect signals of selection is limited. For comparison, we also calculated iHS in British individuals from the 1000 Genomes (GBR) that were included in joint calling and passed subject-level filters (n=90). The iHS values were standardized genome-wide and two-tailed p-values were computed according to the standard normal distribution. We additionally calculated q-values (the minimum False Discovery Rate (FDR) should a test be considered significant) for each test, (Storey and Tibshirani, 2003) and determined p-value significance thresholds at which FDR <0.01 and FDR <0.001 for each population (see Methods). We observed that a number of significant selection signals were shared between the Faroese and British cohorts, which is not unexpected given the relationship between these two populations (Figure 3A, B). The strength of these signals did differ from one population to the other, though this may be due to differences in sample size or changes in selection pressure after the populations diverged. To better identify population-specific signals, we also calculated XPEHH comparing Faroese and British haplotypes, and identified significance cutoffs following the same approach described above (Figure 3C, Sabeti et al., 2007). Any extreme positive values of this statistic indicate longer haplotypes at a focal marker in the Faroese cohort compared to the British cohort, while extreme negative values indicate the reverse. Therefore, positive values are indicative of selection signals in the Faroese cohort. Across both tests, we highlight 20 loci with the most extreme values for these statistics, serving as evidence of positive selection in the Faroese genomes at those loci (Supplementary file 4).

**Figure 3—figure supplement 1.**
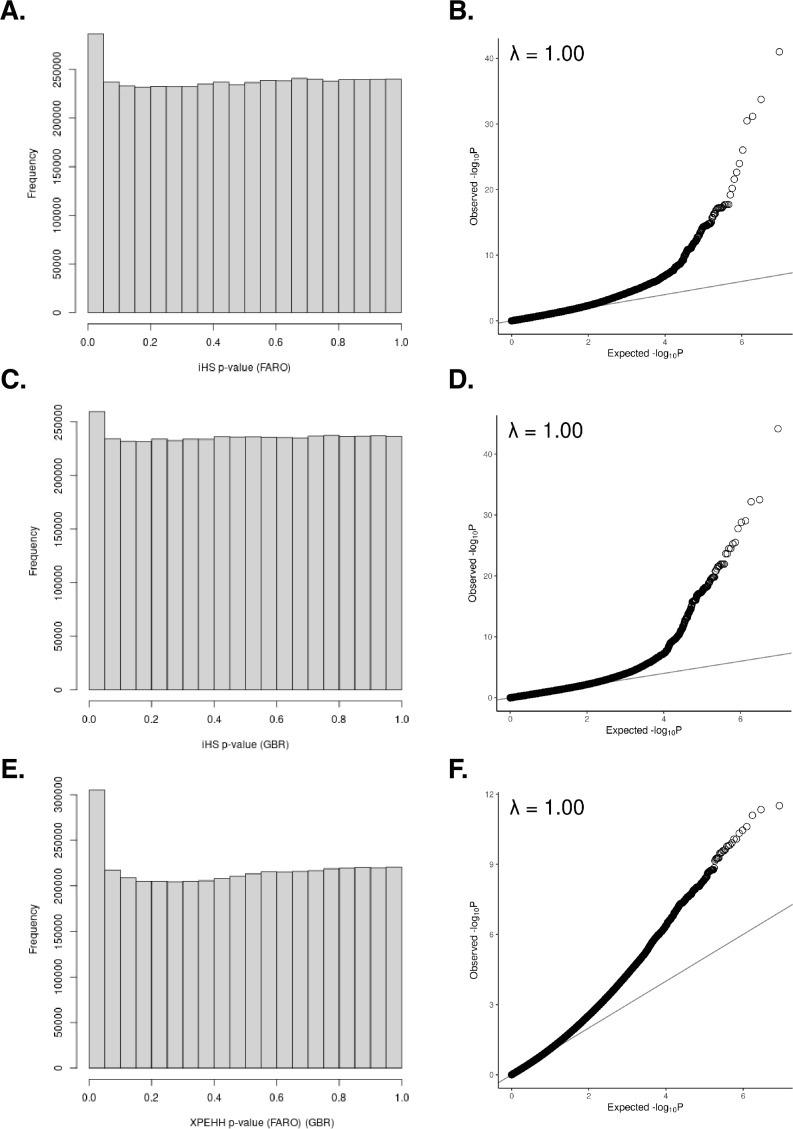
Q-Q plots and p-value histograms for selection statistics. Histograms of p-value distributions for (**A**) integrated haplotype score (iHS) in the Faroese (FARO) haplotypes, (**C**) iHS in the British (GBR) haplotypes, (**E**) cross-population expected haplotype homozygosity (XPEHH) comparing FARO and GBR. Q-Q plots for observed versus expected log-transformed p-values for (**B**) iHS in FARO, (**D**) iHS in GBR (**F**) XPEHH between FARO and GBR. The estimated lambda inflation value is shown in each Q-Q plot.

**Figure 3—figure supplement 2.**
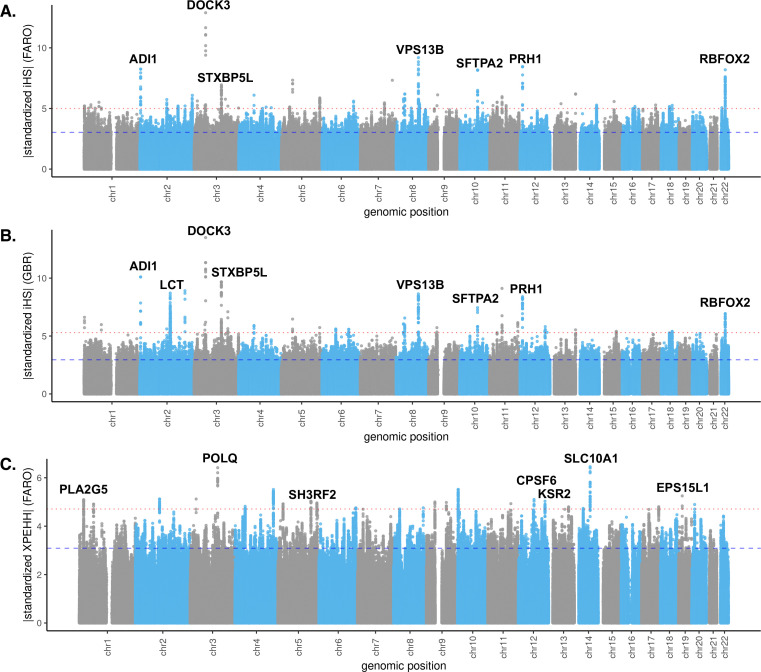
Selection scan results for Faroese and British cohorts. (**A**) Absolute value of the standardized integrated haplotype score (iHS) in the 40 Faroese genomes (FARO). (**B**) Absolute value of the standardized integrated haplotype score (iHS) for 90 British whole genome sequencing (WGS) samples from 1000 Genomes (GBR). (**C**) The standardized cross-population expected haplotype homozygosity (XPEHH) for FARO (all positive values). The top 0.5% of results are indicated by the blue dashed line in each plot, and the top 0.01% of results are indicated by the red dotted line in each plot. Some genes in the top loci are indicated on each plot.

**Figure 4.**
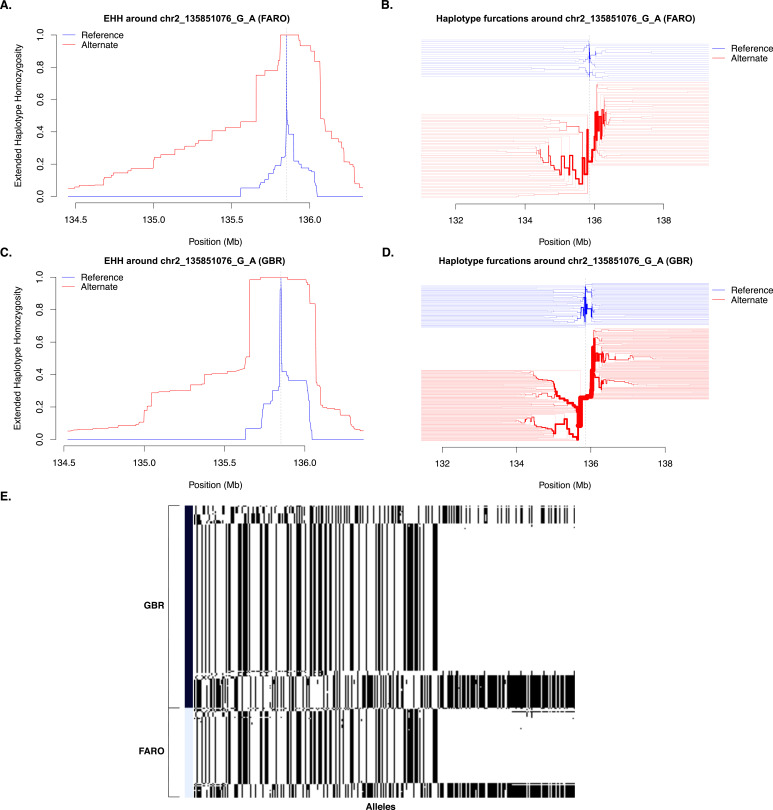
Haplotype visualizations for the LCT/MCM6 locus. (**A**) Decay in Expected Haplotype Homozygosity (EHH) and (**B**) haplotype furcation plot for Faroese (FARO) centered on lactase persistence allele rs4988235; chr2_135851076_G_A. (**C**) Decay in EHH for 1000 Genomes British (GBR) and (**D**) haplotype furcation for GBR centered on the same allele. (**E**) *Haplostrips* visualization of haplotype structure in the region chr2:135677850–135986443. In this panel, columns correspond to segregating alleles, and rows correspond to individuals. In the haplotype furcation plots (panels **B** and **D**), the haplotypes for the reference allele (**G**) are in blue, and those for the alternate allele (**A**) are in red.

One signal that has been consistently observed across northern European populations is in the *LCT*/*MCM6* region, corresponding to positive selection for lactase persistence alleles (Voight et al., 2006; Sabeti et al., 2007; Fan et al., 2016; Williamson et al., 2007). Interestingly, this region showed strong iHS signals (|standardized iHS|>8) in GBR and was considered genome-wide significant in our analysis (minimum *p*=5.95 × 10^–19^, q=7.82 × 10^–14^) (Figure 3B), but the signal is weaker in the Faroese (|standardized iHS|>4) and was not considered significant in our analysis (minimum *p*=3.57 × 10^–6^, q=0.0118) (Figure 3A). To investigate the haplotype structure further, we plotted the decay in expected haplotype homozygosity (EHH) (Sabeti et al., 2002) and haplotype furcation around one of the lactase persistence alleles (rs4988235; chr2_135851076_G_A) for the Faroese and British cohorts using the *rehh* package (Figure 4A–D, Gautier and Vitalis, 2012). The decay and furcation plots are centered around the focal marker, and a furcation occurs when unique haplotypes arise at an allele, similar to a tree splitting into branches. Thicker branches in the furcation plot indicate higher frequency of that haplotype in the population. Significant differences between the furcation patterns for an ‘ancestral’ (reference) and ‘derived’ (alternate) allele correspond to extreme iHS values and therefore are indicative of strong positive selection. For an alternative view of the region, we used *Haplostrips* to visualize the haplotype structure from chr2:135677850–135986443 (Figure 4E, Marnetto and Huerta‐Sánchez, 2017). From these plots, we observed far less diversity on the lactase persistence haplotype in GBR, consistent with a stronger selection signal. This may be explained by shared selection on the ancestral northern European branch followed by either relaxed selection for lactase persistence or population-specific drift in the Faroes after the population split from other northern European groups and settled the archipelago.

**Figure 4—figure supplement 1.**
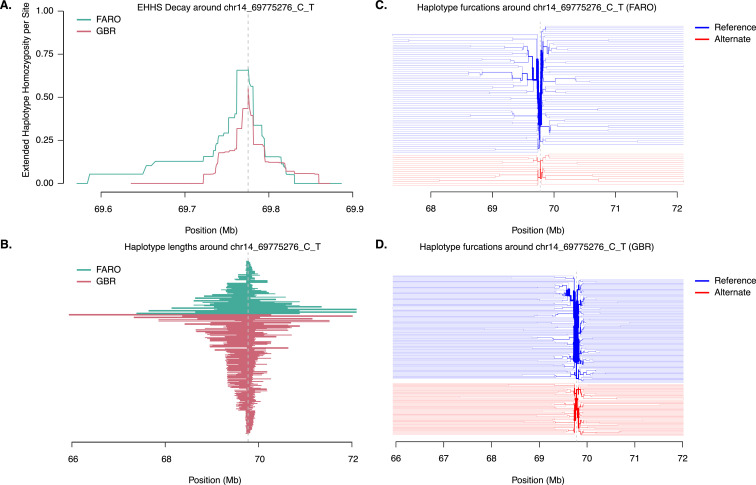
Haplotype visualizations for top XPEHH variant in *SLC10A1*/*SRSF5* locus. (**A**) Decay in Expected Haplotype Homozygosity per Site (EHHS) for chr14_69775276_C_T, comparing Faroese (FARO, teal) and British (GBR, red) haplotypes. (**B**) Lengths for distinct haplotypes spanning chr14_69775276_C_T comparing FARO (teal) and GBR (red). (**C**) haplotype furcation plot for FARO centered on chr14_69775276_C_T (**D**) haplotype furcation for GBR centered on the same allele. In the haplotype furcation plots (panels **C** and **D**), haplotypes for the reference allele (**C**) are in blue, and those for the alternate allele (**T**) are in red.

**Figure 4—figure supplement 2.**
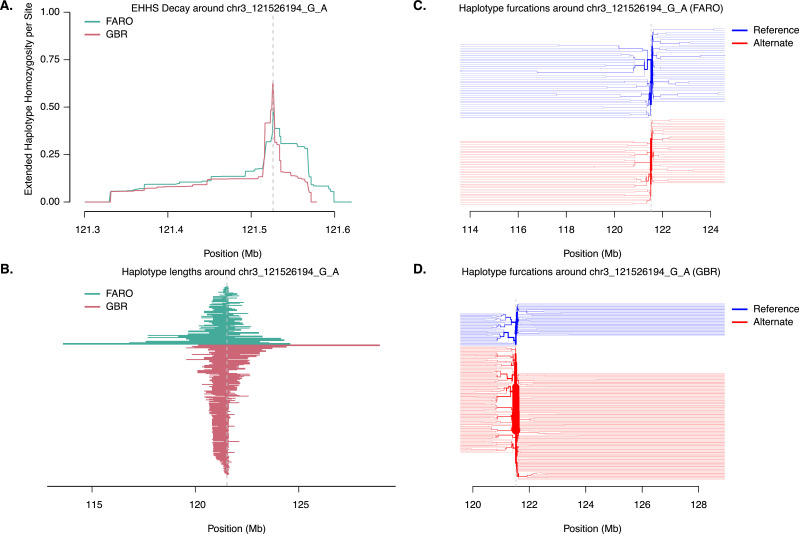
Haplotype visualizations for top XPEHH variant in *POLQ* locus. (**A**) Decay in Expected Haplotype Homozygosity per Site (EHHS) for chr3_121526194_G_A, comparing Faroese (FARO, teal) and British (GBR, red) haplotypes. (**B**) Lengths for distinct haplotypes spanning chr3_121526194_G_A comparing FARO (teal) and GBR (red). (**C**) haplotype furcation plot for FARO centered on chr3_121526194_G_A (**D**) haplotype furcation for GBR centered on the same allele. In the haplotype furcation plots (panels **C** and **D**), haplotypes for the reference allele (**G**) are in blue, and those for the alternate allele (**A**) are in red.

One of the top XPEHH signals in the Faroese WGS cohort included variants in *SLC10A1* (Figure 4—figure supplement 1), a sodium/bile acid transporter that plays a role in circulating bile salts to and from the liver and small intestine for the absorption of dietary fat and fat-soluble vitamins, such as vitamin D (Floerl et al., 2021; Vaz et al., 2015; Ho et al., 2004; Hagenbuch and Meier, 1994). SLC10A1 deficiency has been associated with familial hypercholanemia, or elevated concentrations of bile acids, which can lead to fat malabsorption and vitamin D deficiencies among other secondary health conditions (Vaz et al., 2015; Deng et al., 2016; Liu et al., 2017). Another top XPEHH signal included variants in *POLQ*, encoding for a DNA polymerase which plays a role in DNA repair (Figure 4—figure supplement 2; Ceccaldi et al., 2015; Yoon et al., 2014; Arana et al., 2008; Seki et al., 2003; Wood and Doublié, 2016). *POLQ* has been shown to be involved in various cancers in mice and humans, in particular skin, stomach, lung, breast, and colon cancers (Ceccaldi et al., 2015; Wood and Doublié, 2016; Thomas et al., 2023; Yoon et al., 2019; Pan et al., 2021).

### Fine-scale structure and connections to ancient genomes

Given that early Faroese settlers have documented historical relations to both Northern European Vikings and Northwestern European Celtic communities, we sought to study fine-scale genome-wide ancestry relationships between the sequenced Faroese genomes and publicly available ancient genomes from Iron Age and Viking Age Europe. We downloaded 616 ancient imputed genomes from Allentoft et al., 2024, spanning from the present-day to the late Bronze Age from Europe and focusing specifically on West- and North-Europe, including ancient Faroese genomes (Allentoft et al., 2024). We incorporated these genomes into a combined panel, including our present-day Faroese dataset and used the software HaploNet to infer fine-scale population structure based on patterns of haplotype similarity across the genome (Meisner and Albrechtsen, 2022).

HaploNet identified five ancient sources through unsupervised ancestry estimation. We then used HaploNet to model both ancient and present-day Faroese genomes as composites of any of the five ancestries through supervised ancestry estimation. We used the ancestral haplotype cluster frequency estimates from individuals not found in the Faroe Islands in order to estimate the admixture proportions of these sources in the European mainland. We used these admixture proportions to label the sources based on the locations in the map where these ancestries tend to be maximized in the Iron Age and Viking Age periods. The resulting labels were: ‘Steppe’, ‘East Europe’, ‘Levant and East Mediterranean’, ‘West Europe’, and ‘North Europe.’ For example, the ancestry labeled ‘West Europe’ is maximized in individuals predominantly found in Celtic contexts (e.g. Roman and Iron Age Britain, Iron Age France) while the ancestry ‘North Europe’ is maximized in individuals characteristic of historically Viking or pre-Viking contexts (e.g. Iron Age individuals from Denmark, as well as Viking Age individuals from Denmark, Norway, Sweden, and Estonia). However, we note there is no one-to-one correspondence between archaeological context and genetically inferred ancestry, and that many mainland individuals contain inferred ancestries from diverse sources. Indeed, Margaryan et al., 2020 showed that Viking-context individuals can derive ancestries from multiple Bronze and Iron Age sources across Europe (Margaryan et al., 2020).

We then focused on the frequency of the ancestry sources in the Faroese individuals. We find that the present-day Faroese individuals are predominantly composed of roughly equal proportions of ‘West’ and ‘North Europe’ ancestry, while ‘East Europe’, ‘Steppe’, and ‘Levant’ ancestries are detected at much lower frequencies (40% North Europe, 33.1% West Europe, 12.2% Levant, 8.5% East Europe, 6.2% Steppe). Present-day ancestry proportions are nearly identical to those found in the Faroese ancient samples from the Sandur church site in Sandoy and dated to the 17^th^ or 18^th^ centuries based on their archaeological contexts (37.3% North Europe, 35.6% West Europe, 12.0% Levant, 8.1% East Europe, 7.0% Steppe) (Figure 5—figure supplement 1; Margaryan et al., 2020). Margaryan et al*.* also sequenced a Faroese sample that was excavated from the á Bønhúsfløtu site in the village of Hvalbøur on Suðuroy, and was contextually-dated to be approximately 800 years old. This individual is inferred to be almost entirely composed of ‘West Europe’ ancestry (Figure 5—figure supplement 2).

**Figure 5.**
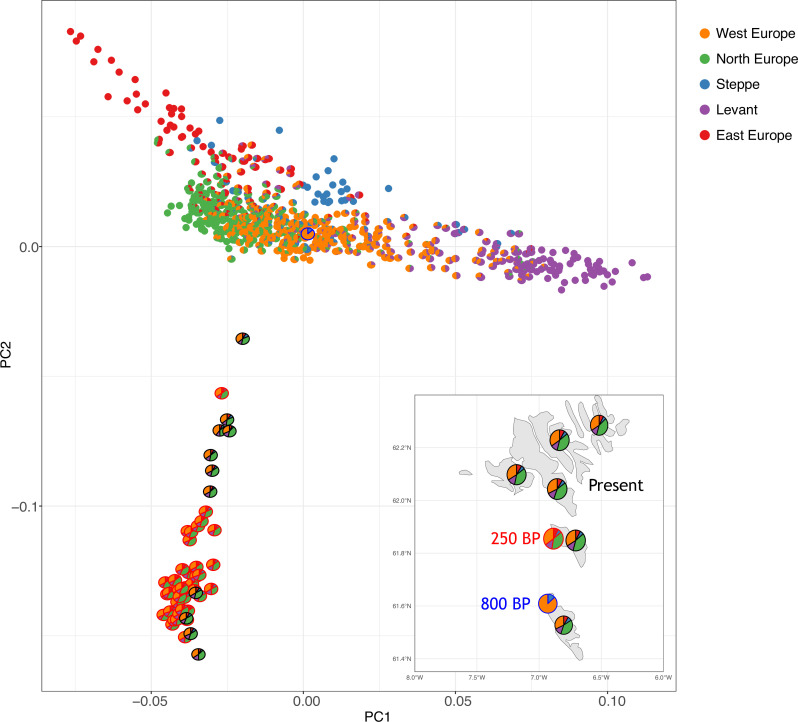
Principal component analysis (PCA) of 616 ancient imputed genomes from Europe and 40 present-day Faroese genomes. Each individual is depicted as a pie chart, showing ancestry proportions estimated using HaploNet. Ancestry proportions for ancient individuals were estimated unsupervised, while those for present-day Faroese individuals were estimated semi-supervised using ancient genomes as references. The five colors represent different ancestral sources: orange for West Europe, green for North Europe, blue for Steppe, purple for the Levant and East Mediterranean, and red for East Europe. The geographical distribution (bottom-right) highlights historical samples (250 years BP) in red, this study’s samples in black, and an 800-year-old individual sample in blue.

We utilized haplotype cluster likelihoods to explore population structure in the same set of samples. When plotting ancient European samples together with the Faroese samples (Figure 5), we observe that present-day Faroese individuals (circled in black) separate from the ancient Europeans along the second principal component (PC2), as do the older Faroese samples from the 17^th^ and 18^th^ centuries (circled in red). This perhaps suggests a bottleneck process that differentiates the 17^th^/18^th^-century and present-day Faroese from the rest of the ancient European samples, in concordance with the above ROH results. Notably, the 800-year-old sample of a Faroese individual with predominantly ‘West Europe’ ancestry does not fall along the Faroese PC2 cline, suggesting that this individual might predate the bottleneck.

**Figure 5—figure supplement 1.**
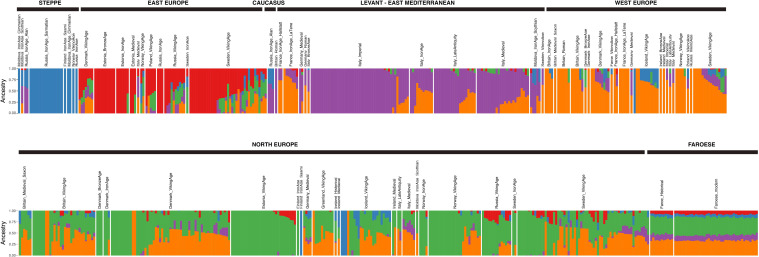
Admixture plot showing proportions for 616 imputed ancient genomes from Europe together with 40 present-day Faroese genomes from this study. Ancient individual groups are categorized based on patterns of IBD clustering as inferred in Allentoft et al., 2024. The plot uses five colors to represent different ancestral sources, which are maximized in individuals in different regions of Europe: orange for ‘West Europe’, green for ‘North Europe’, blue for ‘Steppe’, purple for the ‘Levant and East Mediterranean’, and red for ‘Eastern Europe’.

**Figure 5—figure supplement 2.**
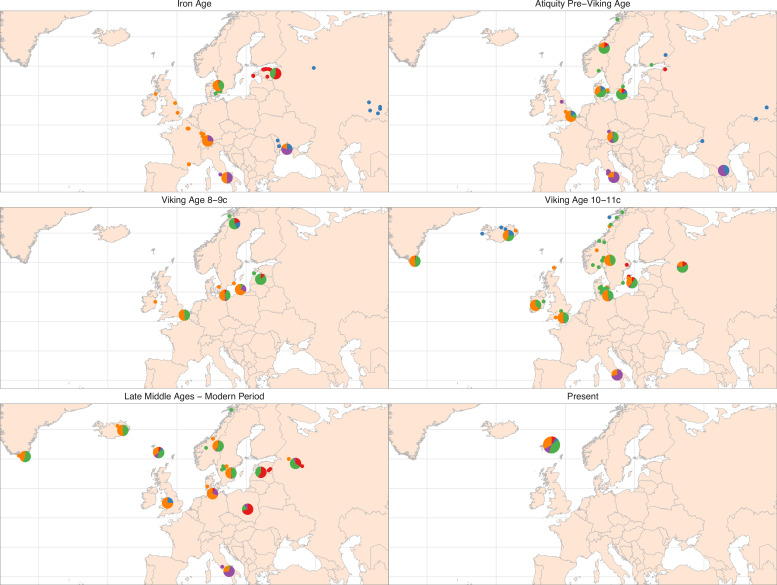
Map illustrating the geographical distribution of ancestry proportions for 616 ancient imputed individuals from Europe and 40 modern Faroese genomes from this study. The map is divided into panels to capture both geographical and temporal variations across Europe. Each pie chart on the map represents admixture proportions with five colors: green, orange, blue, red, and purple maximize Northern European, Celtic, Steppe, Eastern European, and Levant/East Mediterranean ancestries, respectively, reflecting different ancestral sources.

**Figure 5—figure supplement 3.**
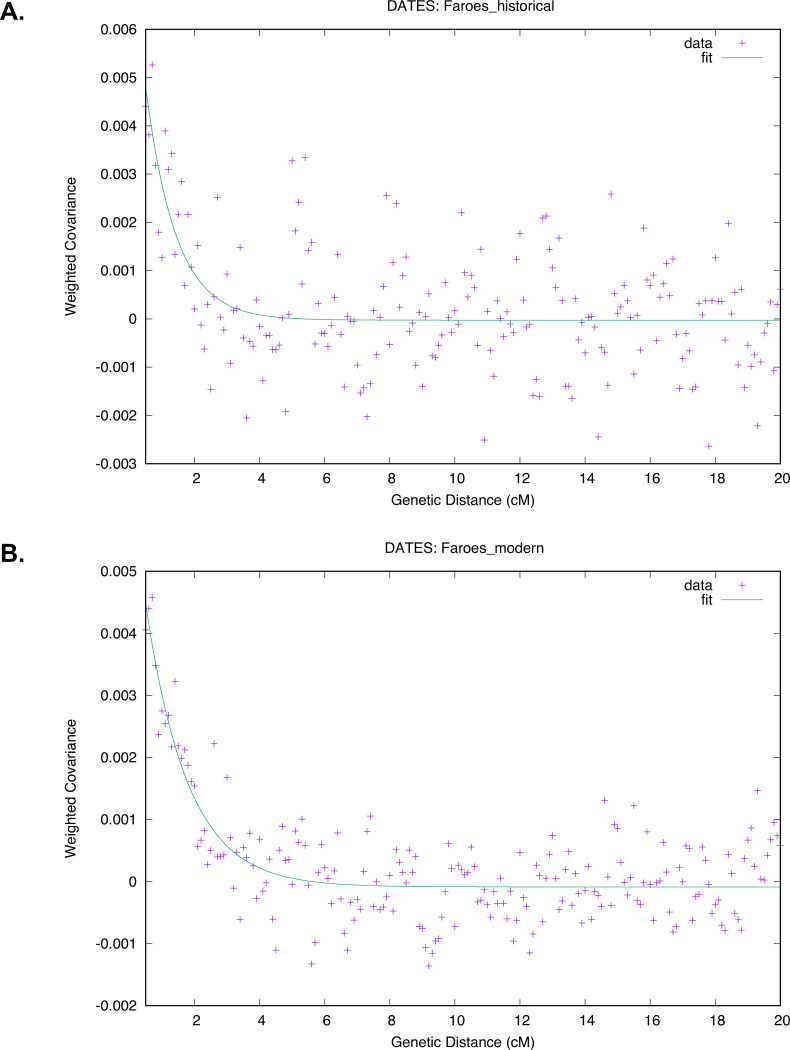
*DATES* estimated ancestry covariance and least squares exponential fit. Plots were output by the *DATES* software for (**A**) 11 historical Faroese individuals (dated to approximately the 18^th^ century) and (**B**) 40 modern Faroese individuals.

We estimated admixture timing for modern (n=40) and ancient (n=11) Faroese individuals using *DATES* with North and West Europe proxy references (Narasimhan et al., 2019). The ancient Faroese had an average admixture estimate of 94.665±58.658 generations prior to the dated age of the samples (~980 BCE; 2681 BCE - 720 CE, assuming 29 year generations) (Figure 5—figure supplement 3A), while present-day Faroese showed more recent admixture at 72.567±15.290 generations in the past (~137 BCE; 581 BCE - 306 CE) (Figure 5—figure supplement 3B). The high standard errors and inconsistency between estimates may reflect confounding due to drift and bottlenecks in the target population (as noted by Narasimhan, Patterson et al.) (Narasimhan et al., 2019) or low differentiation in linkage patterns between the source populations. Additionally, *DATES* assumes a single admixture pulse, but additional waves, particularly in the present-day Faroese, could shift estimates toward the present. In both cases, estimated admixture timing predates Faroese settlement, which likely began in the ninth century CE, (Johnston, 1975; Jorgensen et al., 2004; Young, 1979) but possibly as early as the fourth to sixth centuries CE (Church et al., 2013).

## Discussion

Here, we have presented the first whole-genome sequence data from 40 minimally related individuals from across the Faroe Islands, a North Atlantic founder population. The Faroese have a high prevalence of several diseases in comparison to other European or global populations, several of which have been of particular interest in epidemiological studies of the region (e.g. inflammatory bowel disease, type 2 diabetes, multiple sclerosis) (Leblond et al., 2019; Rasmussen et al., 2014; Passa, 2002; Veyhe et al., 2018; Burisch et al., 2014; Dean et al., 2014; Schwartz et al., 1995; Joensen, 2011; Hammer et al., 2016; Gregersen et al., 2016). Additionally, observational evidence from the FarGen project recruitment data suggest a higher prevalence of ankylosing spondylitis, although follow-up epidemiological studies are required. We investigated the frequency of HLA-B*27 in the Faroe Islands, an allele which has been previously associated with ankylosing spondylitis (AS). We found that despite the observed high prevalence of AS in the Faroe Islands, there was no evidence of increased HLA-B*27 allele frequency compared to other European populations. However, a stop gain variant in *SERPINB10* was among those enriched in the Faroese cohort, and it may contribute to increased AS risk (rs138084090, AF = 2.5%). Rare variant analysis of this gene in the UK Biobank found that an independent stop gain variant in the same gene is nominally associated with increased AS risk (rs145346731, *p*=2.5e-4), which was the most significant phenotypic association for the gene (Karczewski et al., 2022). *SERPINB10* is most highly expressed in neutrophilic metamyelocytes, (George et al., 2024) a cell type with disease relevance to AS (Coletto et al., 2023). *CCDC168*, another gene with a Faroese enriched stop gain variant had highly significant rare variant associations in the UK Biobank with corneal hysteresis and intraocular pressure (rs1361247423, *p*=1.79e-57, *p*=5.21e-13, respectively). Finally, a Faroese enriched stop gain variant in *AGL* identified in this study (rs113994128) has been previously reported as causing glycogen storage disease type IIIA in the Faroe Islands, which is estimated to have the highest prevalence of the disease worldwide (Santer et al., 2001). These findings emphasize the importance of further research into the role that unique genetic variation in the Faroe Islands may play in the incidence of diseases that, while common in the Faroes, have a global burden as well.

We found elevated amounts of the genome contained in long runs of homozygosity (ROHs) compared to other European reference cohorts, including another founder population from Finland. The higher proportion of the Faroese genome contained in these long ROHs suggests a stronger or more recent bottleneck in the Faroese population history. With the second phase of the FarGen study, a larger sample size will facilitate investigation into the timing and severity of the bottleneck(s). Additionally, founder populations, such as the Finnish, have been a common focus for studies of founder events, genetic isolation, and the effects of haplotype sharing on aspects of human health and disease (Norio, 2003; Kerminen et al., 2017; Peltonen et al., 1999; Sabatti et al., 2009). As such, the Faroese population may serve as another useful global reference for studying the influence of demographic history on genetic variation and trait architecture. Long ROHs, in particular, can be enriched for deleterious variation or be of interest in understanding the genetic architecture of health-related traits in human populations (Ceballos et al., 2018; Szpiech et al., 2013). Studying these long ROHs may be relevant for future studies of health outcomes or other traits of interest in the Faroese population.

We also detected several regions under recent positive selection in the population. It is likely that iHS measures selection older than the settlement of the Faroe Islands. Indeed, we found that many of the top selection signals were shared between the Faroese and British cohorts, which is unsurprising given the recent divergence between these two populations and likely reflecting selection that began prior to this divergence. We did find differences in the strength of these signals; for example, there is more diversity on the Faroese *LCT*/*MCM6* lactase persistence haplotypes. The lactase persistence allele rs4988235 (chr2_135851076_G_A) has been inferred to be under strong selection at least until the medieval period in northwestern European groups (Patterson et al., 2022; Burger et al., 2020). Patterson et al., 2022 found that the rate of increase in allele frequency may have slowed in recent periods; however, this does not exclude the possibility of continued or fluctuating selective pressure, as this is consistent with the expected sigmoidal trajectory for an allele under ongoing selection (Haldane, 1927). While the increased diversity on the Faroese lactase persistence haplotypes may be simply explained by population-specific drift, this result could also indicate relaxed selection for lactase persistence alleles after settlement of the Faroe Islands, possibly due to changes in dietary habits in the new environment. The traditional diet of the Faroe Islands consisted of a higher reliance on animal and marine fats, such as sheep tallow, whale blubber, and liver from codfishes, while dairy products, such as milk and cheese, and particularly that from cattle, were more limited in availability (Svanberg, 2021). The selected variant at rs4988235 is at 74% frequency in the modern Faroese cohort, and based on the ancient genomes for which we have available data, we can attest that the allele was present in the Faroe Islands at high frequencies already in the 17^th^/18^th^ centuries (~82%), and imputation further suggests the haplotype containing the allele to at least have been present in the islands 800 years ago. We caveat that the sample size of the historical Faroese (11 individuals) is small and coverage of ancient samples is low, leading to potential errors in imputation. In the absence of selection or drift, we can calculate the expected frequency of an allele in an admixed population as the linear combination of allele frequencies and average ancestry contributions from the sources. Based on the frequency of the rs4988235 variant in proxy sources from the ancient panel, the expected allele frequency in the ancestral population at the time of admixture is approximately 47%. The difference in observed and expected allele frequencies may be due to drift, demography, changes in selection pressure, or a combination of these and other factors. We note limitations in this calculation as the proxy samples may not be good representatives for the true sources at the time of admixture, and there may have been multiple admixture events rather than a single pulse.

We detected selection targets that were specific to the Faroese population using the XPEHH statistic with the British cohort as the comparison population. As XPEHH has the best power to identify alleles that are fixed or approaching fixation in one population but not others, it is unlikely to detect older selection events or incomplete sweeps from shared ancestral populations. One top selection signal is in *POLQ*, which plays a role in DNA repair and various cancers. Without collecting relevant phenotypes or environmental factors, it is difficult to hypothesize what selection pressure may be driving the strong signal in *POLQ*, so this will be an important area of follow-up for future studies. In another top signal, we find *SLC10A1* which plays a role in fat and vitamin D absorption. Positive selection related to differences in dietary fat intake has been hypothesized in many human populations, such as the Inuit population in Greenland (Buckley et al., 2017; Fumagalli et al., 2015). Also situated in a far northern latitude, the Faroese diet is similar to that of the Inuit population, relying on animal and marine fats (Svanberg, 2021; Andersen and Hansen, 2018). The relationship between *SLC10A1* and vitamin D levels may also be relevant, as the northern latitudes of the Faroe Islands and minimal UV exposure can lead to vitamin D deficiency, which has been hypothesized to be a strong selection pressure in populations in extreme latitudes (Hlusko et al., 2018; Nielsen et al., 2017).

Although we hypothesize that these results suggest possible adaptations to environmental pressures of diet or UV exposure in northern latitudes, we cannot draw definitive conclusions based on this current study. It is certainly possible that variants that have risen to high frequencies due to past or ongoing positive selection now play a role in health outcomes in modern populations. For example, the gene *TBC1D4*, which was shown to be under positive selection in the Greenlandic Inuit population likely due to a historical diet low in carbohydrates, has been associated with type 2 diabetes and insulin resistance in the same population (Andersen and Hansen, 2018; Moltke et al., 2014). The prevalences of some diseases enriched in the Faroese population may be related to genomic regions under positive selection. For example, the results of several studies have suggested a role of vitamin D deficiency in the development of multiple sclerosis (Salzer et al., 2012; Sintzel et al., 2018; Laursen et al., 2015). Future studies could involve collection of relevant phenotypes and focus on characterizing selective pressures and fine-mapping targets of selection as have been done in studies that more thoroughly characterized selection signals related to dietary adaptation and UV exposure and their functional consequences in other northern latitude populations (Fumagalli et al., 2015; Andersen and Hansen, 2018; Hlusko et al., 2018; Moltke et al., 2014).

We have inferred ancestry tracts in the present day Faroese genomes that were inherited from ancient populations throughout Europe. We found that present-day Faroese individuals have similar relative ancestry contributions from past ‘North’ and ‘West Europe’ Iron Age populations. The most ancient genome available from the Faroe Islands matches ancestry patterns found in Iron Age West Europe. Admixture could have occurred either via a mixture of the original ‘West Europe’ ancestry with individuals of predominantly ‘North Europe’ ancestry, or by replacement with individuals that were already of mixed ancestry at the time of arrival in the islands (the latter are not uncommon in Viking Age mainland Europe). Our analysis also suggests a bottleneck or a more progressive differentiation process in the islands relative to the mainland, which may postdate the most ancient Faroese genome currently available (approximately 800 years old). The most ancient Faroese sample from Margaryan et al*.* - composed almost entirely of ‘West Europe’ ancestry - is a male individual found in a chapel-site in Suðuroy. Consistent with this, a local legend suggests this site may have been occupied by Irish monks (Arge, 2015). We note that it is difficult to draw conclusions based on a single individual. It is possible that this particular individual moved to the Faroe Islands within their lifetime. More ancient and present-day samples from the islands could shed further light on the history of the Faroese population.

The average admixture timing between ‘North Europe’ and ‘West Europe’ sources (as estimated by *DATES*) pre-dates the settlement of the Faroe Islands (137 BCE - 980 BCE). This is consistent with the low variance in ancestry proportion within the Faroese individuals (both historical and modern), indicating enough time for recombination to break up long ancestry tracts and for global ancestry proportions to reach an equilibrium in the population. That is, these ancestry patterns, combined with the *DATES* estimates, suggest that the present-day Faroese population is most likely descended from already admixed founders who arrived on the islands. Importantly, estimates of admixture timing had high statistical noise, possibly due to several confounding factors including drift, demography, and low differentiation between sources. In particular, it is unclear how the bottleneck history of the Faroese population may affect the performance of *DATES*. In future studies, it will be informative to estimate and simulate the bottleneck size in the Faroese population, and then test the performance of *DATES* on those simulations to confirm whether bottleneck history has affected the empirical estimates of admixture timing. Additionally, it will be important to model single-pulse versus multiple pulses of admixture to determine whether this has resulted in the different estimates for admixture timing in modern and ancient Faroese.

This study focused on population genomic analyses, such as selection scans, population structure, kinship, and ancestry. Given the unique settlement history and genetic architecture of the Faroe Islands, future studies which combine genomic data with relevant phenotype data could provide useful insight into the underlying genetic mechanisms of those traits. In particular, larger-scale genomic studies in the Faroese could investigate genetic risk factors which contribute to the high prevalence of autoimmune and metabolic disease on the islands. This is a focus of the second phase of the FarGen study, which is currently ongoing.

## Methods

### Sample selection and cryptic relatedness

#### FarGen cohort

The participants in this study voluntarily enrolled in the FarGen project (The Faroe Genome Project: https://www.fargen.fo/en/home/). The 1541 subjects are extensively described in Apol et al., 2022. Participant inclusion criteria for the FarGen project are that participants must live in the Faroe Islands or be of Faroese descent. Apol et al*.* report that 96.4% of the participants have between one and four Faroese grandparents. The cohort has a mixed health status composition, with 75% of the participants self-reporting that they have a confirmed diagnosis. Apol et al*.* found that the cohort is somewhat biased in terms of geographical representation, with the capital region being substantially over-represented.

#### Reconstruction of genealogy

The Multi-Generation Register at the Faroese Health Authority describes the ancestry of inhabitants of the Faroe Islands (https://www.fargen.fo/research/multi-generation-registry). The lineages can be traced back to approximately 1650 CE. The register records birth date, parent identities, parents‚ residence at the time of birth, and more. The Legacy Family Tree (https://legacyfamilytree.com) genealogy software is used to manage the digitized records. We reconstructed a genealogical tree of all the individuals in the FarGen cohort by looking up each individual, and recursively looking up their parents until there are no more ancestors. After reconstructing the genealogy of each individual two generations in the past, we discarded any who had fewer than six direct ancestors recorded in the Multi-Generation Registry (two parents and four grandparents). We note that although an individual is recorded in the register, there is no guarantee they were born in the Faroe Islands.

#### Geographical stratification through dialect

We defined six geographical regions of the Faroe Islands as annotated in Figure 1A: Norðoyggjar; Eysturoy and Norðstreymoy; Suðurstreymoy; Vágar and Mykines; Sandoy, Skúvoy, and Stóra Dímun; Suðuroy, and placed individuals within these regions based on birth place. The boundaries of the regions were defined using isoglosses (i.e. boundaries where we see changes in dialect) as described in Þráinsson, 2004. For example, the isogloss for ‘á’, which may be pronounced either as [a:] or [ɔa], separates Norðoyggjar in the north from the rest of the islands.

#### Calculating pairwise kinship coefficients

In order to avoid sequencing highly related samples, we used the large constructed genealogy to account for cryptic relatedness. We calculated pairwise kinship coefficient between every individual using *kinship2* (Sinnwell et al., 2014). This method assumes that the founders (individuals in the pedigree without recorded parents) are unrelated to other founders and each individual founder's parents were not related to each other, which may not always be the case in this population.

#### Sample selection using graph theory

We constructed a relationship graph with nodes representing individuals, and connected two nodes by an edge if their kinship coefficient is above a given threshold as described below. Ideally, we would remove nodes such that all edges are removed, while keeping as many nodes as possible, referred to as the *maximum independent set* problem. Obtaining an exact solution to the maximum independent set is an NP-hard problem (exponential time complexity), making it infeasible for our applications. Instead, we obtained an approximate maximum independent set using an algorithm described in Boppana and Halldórsson, 1992. We performed relatedness pruning with a threshold of 2^−6^ on 1294 FarGen individuals who were not missing a birth region, resulting in 332 individuals. This was the minimum threshold for which we could have enough sampling from each region. From these 332 individuals, we sampled 5–8 from each region, yielding 40 individuals for whole genome sequencing and subsequent analyses.

### Bioinformatics

#### Sequencing and quality control

DNA samples from the 40 selected individuals were sequenced at FarGen (using TruSeq PCR-free libraries on Illumina NextSeq 500 instruments) to an average depth of 19.2 x, ranging from 9.9x to 31.5x per sample. Sequencing QC was investigated with FASTQC, Picard (including CollectWgsMetrics for coverage), and VerifyBamID for contamination.

#### Variant calling and imputation

Reads were processed with Variant Bio's in-house processing pipeline based on the GATK Best Practices (CCDG functional equivalence version). (Regier et al., 2018). Joint genotyping was performed with GATK (version 4.2.0.0) including 714 genomes from the 1000 Genomes Project (503 Europeans, 103 Han Chinese, 108 Yoruba) and followed by VQSR (--truth-sensitivity-filter-level to 99.8 for SNPs and 99.0 for indels). Only PASS variants in GIAB high-confidence regions (~80% of GRCh38) were retained (Olson et al., 2022). Genotypes with GQ ≤20 were filtered (set to missing) and imputation was performed within the full cohort of 754 genomes using Beagle v5.1. Variants enriched in the Faroese cohort and with predicted functional impact (Supplementary file 2) were additionally hard-filtered with VQSLOD >20.

#### HLA typing

We ran HLA*LA (v1.0.3) on the mapped reads to determine HLA types for the 40 Faroese individuals as well as for the GBR, CEU, and FIN reference population individuals from 1000 Genomes (Dilthey et al., 2019). Benchmarking the method on 1000 Genomes Project data, where HLA types are known, we estimate overall HLA-B typing accuracy at 93.4%, 88.8%, and 89.9% for the GBR (N=91), CEU (N=99), and FIN (N=99) reference populations. Accuracy of B*27 detection specifically is 100% (N=10), 83.3% (N=6) and 100% (N=15) based on these three reference cohorts, respectively, with one B*27:05 allele misidentified as B*27:26 in CEU. Supplementary file 3 contains counts, mean quality scores, and frequencies of all HLA-B alleles detected among the 40 Faroese as well as the GBR, CEU, and FIN reference populations.

### Population genetics

#### Individual and variant-level filtering

Beginning with a total of 21,837,577 variants and 754 individuals, we applied various individual-level and variant-level quality control filters for downstream analyses. We filtered 8 individuals with mismatched sex based on genetics and reported information. We additionally filtered five individuals that were, for any of PCs 1–10, further than seven standard deviations from the mean. We then filtered 481,403 variants that were not in GIAB high-confidence regions, had MQ rank sum not equal to zero or failed gnomAD v3 QC (Karczewski et al., 2020). We removed 3627 variants with a minor allele count of less than 1 after individual-level filters were applied. We removed 695,947 variants that were not autosomal. This final dataset included 20,656,600 variants and 741 individuals.

#### Selection scans

iHS and XPEHH were calculated using the *hapbin* software (https://github.com/evotools/hapbin; Maclean et al., 2019) with option –max-extend 1,000,000 and --minmaf 0.05 (Maclean et al., 2015). All other options were set to default. To compute P-values, we used the method by Fariello et al., 2013, exploiting the fact that detectable regions under strong selection affect a small portion of the genome (Fariello et al., 2013). For both statistics, values were standardized genome-wide in 2% allele frequency bins, as allele frequency is correlated with allele age and, therefore, haplotype length (Voight et al., 2006; Sabeti et al., 2007). We first computed outlier-robust mean and standard deviation with the rlm() function from the MASS package in *R*, to reduce the influence of outliers (Fariello et al., 2013; Venables and Ripley, 2002). The standardized values of these summary statistics represent z-scores. We calculated two-tailed p-values using these z-scores, giving the probability that we observe these data by chance compared to null expectations for the standard normal distribution. Q-Q plots and histograms of p-values for each statistic are provided in the Supplementary Materials (Figure 3—figure supplement 1). For each summary statistic distribution, we also calculated the p-value cutoffs that correspond to a False Discovery Rate (FDR) of less than 0.01 and 0.001, using the q-value *R* package (https://github.com/StoreyLab/qvalue) (Storey et al., 2023; Storey and Tibshirani, 2003). We additionally include the empirical standardized values for each statistic (Figure 3—figure supplement 2).

EHH decay plots and haplotype furcations for the *LCT* locus were calculated using the *rehh R* package (https://cran.r-project.org/package=rehh) (Gautier and Vitalis, 2012) and visualized the haplotype structure of the genomic region chr2:135677850–135986443 using *haplostrips* and plot option -S 3 (https://bitbucket.org/dmarnetto/haplostrips) (Marnetto, 2023; Marnetto and Huerta‐Sánchez, 2017).

#### Kinship and runs of homozygosity

The kinship matrix in the WGS cohort was calculated using the *popkin* software (https://github.com/StoreyLab/popkin) (Ochoa, 2026; Ochoa and Storey, 2021; Ochoa and Storey, 2019a; Ochoa and Storey, 2019b). We restricted the analyses to biallelic SNPs with a minor allele frequency of at least 0.01 in at least one subpopulation (i.e. YRI, CHB, FIN, CEU, GBR, TSI, IBS, or FARO), resulting in a dataset of 15,206,409 variants. When calculating the kinship matrix for the Faroese WGS cohort only, we used the *rescale_kinship()* function, which will change the most recent common ancestor and give different absolute values, but the overall relationship structure in the subpopulation remains the same. Using the same data set, we calculated runs of homozygosity (ROHs) for each individual using *bcftools/RoH* (Narasimhan et al., 2016). ‘Short’ ROHs were classified as ROH less than or equal to 300 kb, ‘medium’ as greater than 300 kb and less than or equal to 1 Mb, and ‘long’ as greater than 1 Mb.

#### Fine-scale structure estimation using ancient genomes

A panel of 616 imputed ancient genomes from Allentoft et al., 2024 (downloaded from https://doi.org/10.17894/ucph.d71a6a5a-8107-4fd9-9440-bdafdfe81455), representing individuals from several European regions (southern Europe, western Europe, northern Europe, eastern Europe, and central Europe) was used for analyses (Allentoft et al., 2024). Only samples that were estimated to be no older than 3000 years old were used. Out of these ancient samples, 12 were excavated in the Faroe Islands, 11 of them are historical samples dated to approximately 250 years old, and 1 of them is dated to be approximately 800 years old (Margaryan et al., 2020). The sample location, approximate age, and sources for these samples are listed in Supplementary file 5. To consolidate the two panels, we first performed a liftover of the ancient genome VCF files to the GRCh38 reference genome. Following this, we applied quality filters to the dataset (bi-allelic sites MAF >0.05 and imputation INFO ≥0.5).

We used HaploNet - a neural network-based method for performing window-based haplotype clustering across the genome - for fine-scale population structure inference on the combined panel. HaploNet uses a hidden Markov model to find an optimal window-based local ancestry ‘painting’ across a genome, given estimated haplotype cluster likelihoods, haplotype cluster frequencies, and global ancestry proportions (Scheet and Stephens, 2006). The Faroese panel’s haplotype frequencies are very homogeneous and highly differentiated from mainland Europeans. For this reason, under an initial round of unsupervised ancestry estimation, we found that the Faroese individuals captured a major component at first split (K=2). We, therefore, implemented and utilized a semi-supervised ancestry estimation feature in HaploNet (Meisner and Albrechtsen, 2022). We performed haplotype clustering in non-overlapping windows of 512 SNPs, and we used the resulting haplotype cluster likelihoods to perform principal component analysis (PCA) and estimate both global and local ancestry in the Faroese individuals.

We performed global ancestry estimation in HaploNet using its EM algorithm to find the maximum likelihood estimates using only ancient European individuals (excluding the Faroese individuals) (Meisner and Albrechtsen, 2022). We used the EM algorithm a second time to estimate the ancestry proportions (Q matrix) in the Faroese individuals. The estimated haplotype cluster frequencies (F matrix) were kept fixed, which means that the semi-supervised approach can be seen as modeling the Faroese individuals using inferred haplotype clusters from the ancient European individuals.

We estimated the average timing of admixture in modern and ancient Faroese individuals using the *DATES* software (Narasimhan et al., 2019). We selected reference individuals from the ancient panel who were maximized for North Europe (n=64) and West Europe (n=41) ancestry, respectively. We obtained separate estimates for the admixture timing in the 11 historical Faroese individuals dated to approximately the 18^th^ century and the 40 modern individuals from the FarGen project. We used the following recommended default options for optimal performance: binsize = 0.001, maxdis = 1.0, jackknife = YES, qbin = 10, runfit = YES, afffit = YES, lovalfit = 0.45.

## Data Availability

Custom scripts for analyses described in this manuscript can be found on GitHub (https://github.com/olavurmortensen/wgs_selection, Mortensen, 2020). Additional analyses in this study utilized publicly available software and pipelines as described and cited in the Methods. Variant-level summary statistics and genome-wide selection scan results for iHS and XPEHH are available for research via Zenodo (https://doi.org/10.5281/zenodo.19475278). Individual-level genetic and associated metadata are not publicly available due to participant consent restrictions. These data carry a risk of re-identification, particularly in a small population, and are therefore accessible only for approved research under controlled access conditions. Genetic and meta data from this study is stored at the Faroese Health Authority. Researchers will be granted access to de-identified genetic data and metadata, provided that a research proposal describing the intended use of the data is submitted and the project protocol has been approved by the Faroese Scientific Ethical Committee and a template material/data transfer agreement has been signed with the Faroese Health Authority in compliance with GDPR (see Gregersen et al., 2021). Depending on the scope of the proposed research, additional participant re-consent may be required. Requests should be made to Noomi O. Gregersen (noomi@fargen.fo). All data are available for non-commercial use only. The following dataset was generated: HamidI Refoyo-MartínezA LydersenL EmdeAK HendershottM ApolK AndorsdóttirG MeisnerJ WasikK RacimoF CastelS GregersenN 2026Faroese Whole Genomes Provide Insight into Ancestry and Recent SelectionZenodo10.5281/zenodo.19475278PMC1327174442300124 The following previously published dataset was used: AllentoftME SikoraM WillerslevE 2023Genotype data from Population Genomics of Postglacial Western EurasiaERDA10.17894/ucph.d71a6a5a-8107-4fd9-9440-bdafdfe81455

## Additional files

Supplementary file 1. Whole genome sequencing quality control metrics. Unique identifier (subject), sex, region, mean WGS depth (mean_depth), mean depth on X chromosome (mean_depth_chrX), mean depth on Y chromosome (mean_depth_chrY), median insert size (insert_median), mean absolute deviation of insert size (insert_mad), contamination estimate (contamination), and number of genotype calls with GQ >20 (non_imputed_calls). The Faroes region labels are: VM = Vágar and Mykines; SR = Suðuroy; SM = Suðurstreymoy; SD = Sandoy, Skúvoy, Stóra Dímun; NG = Norðoyggjar; and EN = Eysturoy og Norðstreymoy.

Supplementary file 2. Faroese putatively functional alleles. Variants in the joint call set with CADD >30 and at least two minor alleles observed in Faroese individuals, and no minor alleles observed in Finnish or Northern European reference individuals. Allele frequencies in Faroese individuals (af_FARO), allele frequencies in 1000 Genomes British (af_GBR), Central European (af_CEU), and Finnish (af_FIN), and allele frequencies in 1000 G Europeans (af_1000 G_EUR) and gnomAD (af_GNOMAD) references.

Supplementary file 3. HLA-B allele frequencies. Counts of HLA-B alleles out of the total 80 haplotypes and mean quality scores in the Faroese WGS cohort, as well as HLA-B allele frequencies in three 1000 Genomes European cohorts (British [GBR], Central European [CEU], and Finnish [FIN]). HLA genotypes were determined using HLA*LA.

Supplementary file 4. Genomic regions with strong signals of positive selection. Genomic regions for the top 10 standardized iHS and XPEHH values in the Faroese WGS cohort. We list the number of variants and all variant IDs in these regions which reach the p-value threshold corresponding to FDR <0.01 (iHS: *p*<2.72 × 10^–6^; XPEHH: *p*<2.35 × 10^–6^). The regions are defined by the first and last variant position with a p-value below the cutoff for each particular statistic, as indicated in the ‘statistic’ column. Protein-coding gene name(s), if any, with annotated variants reaching these thresholds are provided. We additionally provide the maximum absolute value of the given statistic (‘max_statistic’), its corresponding p-value and q-value (i.e. the minimum FDR when that particular test is considered significant), and the allele frequencies in the Faroese and British cohorts for the variant(s) with the maximum value. The variant(s) with the maximum value for each region are highlighted in bold font.

Supplementary file 5. Admixture and ancient ancestry samples. Region, country, approximate age, publication source, and group label for ancient ancestry samples included in the ADMIXTURE and HaploNet analyses.

MDAR checklist
