# Supplementary material for: Faroese whole genomes provide insight into ancestry and recent selection
Source: eLife. 2026 Jun 16;14:RP107428. doi: 10.7554/eLife.107428 (PMC13271744; doi:10.7554/eLife.107428)
Supplement: Peer review history

# Editor report: eLife Assessment

**DOI:** 10.7554/eLife.107428.3.sa0

**Authors:** Detlef Weigel

**Affiliations:** Max Planck Institute for Biology Tübingen Germany

**Keywords:** Convincing, Useful

This **useful** study analyzes demographic history and selection using whole-genome sequencing data from 40 Faroese individuals, generating results of value beyond the study region. The analyses are **convincing**, and revisions have satisfactorily addressed prior concerns, including clarification of selection analyses and expanded discussion of population structure and admixture timing. While a more fine-scale reconstruction of demographic history could still yield more insights, and access restrictions on individual-level data continue to limit broader reuse, the provision of summary statistics partially mitigates this constraint.

# Referee report: Reviewer #1 (Public review)

**DOI:** 10.7554/eLife.107428.3.sa1

Summary:

The paper reports an analysis of whole-genome sequence data from 40 Faroese. The authors investigate aspects of demographic history and natural selection in this population. The key findings are that Faroese (as expected) have a small population size and are broadly of Northwest European ancestry. Accordingly, selection signatures are largely shared with other Northwest European populations although the authors identify signals that may be specific to the Faroes. Finally they identify a few predicted deleterious coding variants that may be enriched in the Faroes.

Strengths:

The data are appropriately quality controlled and appear to be high quality. Some aspects of Faroese population history are characterized - in particular, the relatively (compared to other European populations) high proportion of long runs of homozygosity, which may be relevant for disease mapping of recessive variants. The selection analysis is presented reasonably, although as the authors point out, many aspects, for example differences in iHS, can reflect differences in demographic history or population-specific drift and thus can't reliably be interpreted in terms of differences in the strength of selection.

Weaknesses:

The main limitations of the paper are as follows:

(1) The data are not available. I appreciate that (even de-identified) genotype data cannot be shared, however, that does substantially reduce the value of the paper. I appreciate the authors sharing summary statistics for the selection scan.

(2) The insight into the population history of the Faroes is limited, relative to what is already known (i.e. they were settled around 1200 years ago, by people with a mixture of Scandinavian and British ancestry, have a small effective population size, and any admixture since then comes from substantially similar populations). It's obvious, for example that the Faroese population has a smaller bottleneck than, say, GBR.

More sophisticated analyses (for example, ARG-based methods, or IBD or rare variant sharing) would be able to reveal more detailed and fine-scale information about the history of the populations that is not already known. PCA, ADMIXTURE and HaplotNet analysis are broad summaries, but the interesting questions here would be more specific to the Faroes, for example, What are the proportions of Scandinavian vs Celtic ancestry? What is the date and extent of sex bias (as suggested by the uniparental data) in this admixture? I think that it a bit of a missed opportunity not to address these questions.

(3) I don't really understand the rationale for looking at HLA-B allele frequencies. The authors write that "Observational evidence from the FarGen project recruitment data suggest that ankylosing spondylitis (AS) may be at a higher prevalence in the Faroe Islands". But nothing beyond that. So there's no evidence (certainly no published evidence) that AS is more prevalent, and hence nothing to explain with the HLA allele frequencies? This section seems preliminary.

# Referee report: Reviewer #2 (Public review)

**DOI:** 10.7554/eLife.107428.3.sa2

In this paper, Hamid et al present 40 genomes from the Faroe Islands. They use these data (a pilot study for an anticipated larger-scale sequencing effort) to discuss the population genetic diversity and history of the sample, and the Faroes population. I think this is an overall solid paper; it is overall well-polished and well-written. It is somewhat descriptive (as might be expected for an explorative pilot study), but does make good use of the data.

The data processing and annotation follows a state-of-the-art protocol, and at least I could not find any evidence in the results that would pinpoint towards bioinformatic issues having substantially biased some of the results, and at least preliminary results lead to the identification of some candidate disease alleles, showing that small, isolated cohorts can be an efficient way to find populations with locally common, but globally rare disease alleles.

I also enjoyed the population structure analysis in the context of ancient samples, which gives some context to the genetic ancestry of Faroese, although it would have been nice if that could have been quantified, and it is unfortunate that the sampling scheme effectively precludes within-Faroes analyses.

Comments on the revision:

I appreciate the authors' detailed and thoughtful response to my review. They have addressed all my concerns to my satisfaction and I have no additional comments.

# Author comment: Author response

**DOI:** 10.7554/eLife.107428.3.sa3

**Authors:** Iman Hamid, Ólavur Mortensen, Alba Refoyo-Martínez, Leivur N Lydersen, Anne-Katrin Emde, Melissa Hendershott, Katrin D Apol, Guðrið Andorsdóttir, Jonas Meisner, Kaja A Wasik, Fernando Racimo, Stephane E Castel, Noomi O Gregersen

**Affiliations:** Variant Bio Inc. Seattle United States; National Hospital of the Faroe Islands Tórshavn Faroe Islands; University of Copenhagen Copenhagen Denmark; National Hospital of the Faroe Islands Tórshavn Faroe Islands; Variant Bio, Inc. Seattle United States; Variant Bio Inc. Seattle United States; National Hospital of the Faroe Islands Tórshavn Faroe Islands; National Hospital of the Faroe Islands Tórshavn Faroe Islands; Copenhagen University Hospital Copenhagen Denmark; Variant Bio Inc. Seattle United States; University of Copenhagen Copenhagen Denmark; Variant Bio Inc. Seattle United States; National Hospital of the Faroe Islands Tórshavn Faroe Islands

The following is the authors’ response to the original reviews.

We thank the reviewers for their thoughtful comments and constructive suggestions. We describe how we have addressed each point below and are grateful for the guidance on areas where our work could be clarified or expanded. In particular, we note the following:

Selection scan summary statistics: In our revised manuscript, we have included summary statistics from the selection scans. We believe this addition will enhance transparency and provide additional context for readers.

Reporting of outliers: As highlighted by the editor, the reviewers expressed differing views on the most appropriate way to report outliers. To provide a comprehensive and balanced presentation, we now report both the empirical selection statistics and the corresponding converted p-values in either the main text or supplement, and both outputs are also provided in the full summary files. This dual approach will allow readers to fully interpret the results under both perspectives.

Expanded discussion of admixture timing and population structure: We have carefully considered the reviewers' suggestions to incorporate additional descriptions of population structure or demographic analyses, and have done so in our revisions where possible. These changes strengthen the rigor and clarity of the analyses.

**Public Reviews:**

**Reviewer #1 (Public review):**

Summary:

The paper reports an analysis of whole-genome sequence data from 40 Faroese. The authors investigate aspects of demographic history and natural selection in this population. The key findings are that the Faroese (as expected) have a small population size and are broadly of Northwest European ancestry. Accordingly, selection signatures are largely shared with other Northwest European populations, although the authors identify signals that may be specific to the Faroes. Finally, they identify a few predicted deleterious coding variants that may be enriched in the Faroes.

Strengths:

The data are appropriately quality-controlled and appear to be of high quality. Some aspects of the Faroese population history are characterized, in particular, by the relatively (compared to other European populations) high proportion of long runs of homozygosity, which may be relevant for disease mapping of recessive variants. The selection analysis is presented reasonably, although as the authors point out, many aspects, for example differences in iHS, can reflect differences in demographic history or population-specific drift and thus can't reliably be interpreted in terms of differences in the strength of selection.

Weaknesses:

The main limitations of the paper are as follows:

(1) The data are not available. I appreciate that (even de-identified) genotype data cannot be shared; however, that does substantially reduce the value of the paper. Minimally, I think the authors should share summary statistics for the selection scans, in line with the standard of the field.

We agree with the reviewer that sharing the selection scan results is important, so we have now made the selection scan summary statistics publicly available, and clearly lay out the guidelines and research questions for which the data can be accessed in our Data Availability statement.

(2) The insight into the population history of the Faroes is limited, relative to what is already known (i.e., they were settled around 1200 years ago, by people with a mixture of Scandinavian and British ancestry, have a small effective population size, and any admixture since then comes from substantially similar populations). It's obvious, for example, that the Faroese population has a smaller bottleneck than, say, GBR.

More sophisticated analyses (for example, ARG-based methods, or IBD or rare variant sharing) would be able to reveal more detailed and fine-scale information about the history of the populations that is not already known. PCA, ADMIXTURE, and HaplotNet analysis are broad summaries, but the interesting questions here would be more specific to the Faroes, for example, what are the proportions of Scandinavian vs Celtic ancestry? What is the date and extent of sex bias (as suggested by the uniparental data) in this admixture? I think that it is a bit of a missed opportunity not to address these questions.

We clarify that we did quantify the proportions of various ancestry components as estimated by HaploNet in main text Figure 5 and supplemental figures S6 and S7. To better highlight this result, we now also include the average global ancestry of the various components in the Main Text - Results - Fine-Scale Structure and Connections to Ancient Genomes.

We agree that more fine-scale demographic analyses would be informative. We now additionally provide an estimation of the admixture date in the Main Text - Results - Fine-Scale Structure and Connections to Ancient Genomes and discussion using the DATES software which is optimized for ancient genomes.

We have encountered problems with using different standard date estimation software, including DATES, which give very inconsistent and unstable results. As we note in our text, we suspect this might be due to the strong bottleneck experienced in the history of the Faroe Islands, low LD differentiation between the source populations, or multiple pulses of admixture, which may be breaking one or more of the assumptions of these methods. Assessing the limitations of these methods is beyond the scope of this current manuscript; however, we will continue working on this problem for future studies, possibly using simulations to assess where the problem might be. We recognize that our relatively small sample size places limits on the fine-scale demographic analyses that can be performed. We are addressing this in ongoing work by generating a larger cohort, which we hope will enable more detailed inference in the future.

(3) I don't really understand the rationale for looking at HLA-B allele frequencies. The authors write that "ankylosing spondylitis (AS) may be at a higher prevalence in the Faroe Islands (unpublished data), however, this has not been confirmed by follow-up epidemiological studies". So there's no evidence (certainly no published evidence) that AS is more prevalent, and hence nothing to explain with the HLA allele frequencies?

We agree that no published studies have confirmed a higher prevalence of ankylosing spondylitis (AS) in the Faroe Islands. Our recruitment data suggest that AS might be more common than in other European populations, but we understand that this is only based on limited, unpublished observations and what we are hearing from the community. We emphasized in our original manuscript that this is based on observational evidence from the FarGen project. However, as this reviewer pointed out, we can be more clear that this prevalence has not been formally studied.

In revision, we clarify in the Main Text - Results - HLA-B Allele Frequencies and Discussion that our recruitment data suggest a higher prevalence of AS may be possible, but more formal epidemiological studies are needed to confirm this observation. The reason we study HLA-B allele frequencies is to see if the genetic background of the Faroese population could help explain this possible difference, since HLA-B*27 is already known to play a strong role in AS.

**Reviewer #2 (Public review):**

In this paper, Hamid et al present 40 genomes from the Faroe Islands. They use these data (a pilot study for an anticipated larger-scale sequencing effort) to discuss the population genetic diversity and history of the sample, and the Faroes population. I think this is an overall solid paper; it is overall well-polished and well-written. It is somewhat descriptive (as might be expected for an explorative pilot study), but does make good use of the data.

The data processing and annotation follows a state-of-the-art protocol, and at least I could not find any evidence in the results that would pinpoint towards bioinformatic issues having substantially biased some of the results, and at least preliminary results lead to the identification of some candidate disease alleles, showing that small, isolated cohorts can be an efficient way to find populations with locally common, but globally rare disease alleles.

I also enjoyed the population structure analysis in the context of ancient samples, which gives some context to the genetic ancestry of Faroese, although it would have been nice if that could have been quantified, and it is unfortunate that the sampling scheme effectively precludes within-Faroes analyses.

We note that although the ancestry proportions were not originally specified in the main text, we did quantify ancestry proportions in the modern Faroese individuals and other ancient samples, and we visualized these proportions in Figure 5 and Supplementary Figures S6 and S7. As stated in our response to Reviewer #1, in our revisions, we now more clearly state the average global ancestry of the various components in the Main Text - Results - Fine-Scale Structure and Connections to Ancient Genomes.

I am unfortunately quite critical of the selection analysis, both on a statistical level and, more importantly, I do not believe it measures what the authors think it does.

Major comments:

(1) Admixture timing/genomic scaling/localization:

As the authors lay out, the Faroes were likely colonized in the last 1,000-1,500 years, i.e., 40-60 generations ago. That means most genomic processes that have happened on the Faroese should have signatures that are on the order of ~1-2cM, whereas more local patterns likely indicate genetic history predating the colonization of the islands. Yet, the paper seems to be oblivious to this (to me) fascinating and somewhat unique premise. Maybe this thought is wrong, but I think the authors miss a chance here to explain why the reader should care beyond the fact that the small populations might have high-frequency risk alleles and the Faroes are intrinsically interesting, but more importantly, it also makes me think it leads to some misinterpretations in the selection analysis.

See response to point #3

(2) ROH:

Would the sampling scheme impact ROH? How would it deal with individuals with known parental coancestry? As an example of what I mean by my previous comment, 1MB is short enough in that I would expect most/many 1MB ROH-tracts to come from pedigree loops predating the colonization of the Faroes. (i.e, I am actually quite surprised that there isn't much more long ROH, which makes me wonder if that would be impacted by the sampling scheme).

The sampling scheme was designed to choose 40 Faroese individuals that were representative of the different regions and were minimally related. There were no pairs of third-degree relatives or closer (pi-hat > 0.125) in either the Faroese cohort or the reference populations. It is possible that this sampling scheme would reduce the amount of longer ROHs in the population, but we should still be able to see overall patterns of ROH reflective of bottlenecks in the past tens of generations. Additionally, based on this reviewer's earlier comment, 1 Mb ROHs would still be relevant to demographic events in the last 40-60 generations given that on average 1 cM corresponds to 1 Mb in humans, though we recognize that is not an exact conversion.

That said, the “sum total amount of the genome contained in long ROH” as we described in the manuscript includes all ROHs greater than 1Mb. Although we group all ROHs longer than 1Mb into one category in Main Text Figure 2, we now additionally provide the distribution in ROH lengths across all individuals for each cohort in a new Supplemental Figure S3. As this plot shows, there certainly are ROHs longer than 1Mb in the Faroese cohort, and on average there is a higher proportion of long ROH particularly in the 5-15 Mb range in the Faroese cohort relative to the other cohorts. As the reviewer points out, these longer ROHs are possibly indicative of a more recent or stronger bottleneck in the Faroes relative to the comparison cohorts. We highlight this result in Main Test - Results - Population Structure and Relatedness.

(3) Selection scan:

We are talking about a bottlenecked population that is recently admixed (Faroese), compared to a population (GBR) putatively more closely related to one of its sources. My guess would be that selection in such a scenario would be possibly very hard to detect, and even then, selection signals might not differentiate selection in Faroese vs. GBR, but rather selection/allele frequency differences between different source populations. I think it would be good to spell out why XP-EHH/iHS measures selection at the correct time scale, and how/if these statistics are expected to behave differently in an admixed population.

The reviewer brings up good points about the utility of classical selection statistics in populations that are admixed or bottlenecked, and whether the timescale at which these statistics detect selection is relevant for understanding the selective history of the Faroese population. We break down these concerns separately.

(1) Bottlenecks: Recent bottlenecks result in higher LD within a population. However, demographic events such as bottlenecks affect global genomic patterns while positive selection is expected to affect local genomic patterns. For this reason, iHS and XP-EHH statistics are standardized against the genome-wide background, to account for population-specific demographic history.

(2) Admixture: The term “admixture” has different interpretations depending on the line of inquiry and the populations being studied. Across various time and geographic scales, all human populations are admixed to some degree, as gene flow between groups is a common fixture throughout our history. For example, even the modern British population has “admixed” ancestry from North / West European sources as well, dating to at least as recently as the Medieval & Viking periods (Gretzinger et al. 2022, Leslie et al. 2015), yet we do not commonly consider it an “admixed” population, and we are not typically concerned about applying haplotype-based statistics in this population. This is due to the low divergence between the source populations. In the case of the Faroe Islands, we believe admixture likely occurred on a similar timescale or even earlier, based on the DATES estimates. We see low variance in ancestry proportions estimated by HaploNet, both from the historical Faroese individuals (dated to 260 years BP) and the modern samples. This indicates admixture predating the settlement of the Faroe Islands, where recombination has had time to break up long ancestry tracts and the global ancestry proportions have reached an equilibrium. That is, these ancestry patterns suggest that the modern Faroese are most likely descended from already admixed founders. In the original manuscript, we mentioned this as a likely possibility in the Main Text - Discussion: “This could have occurred either via a mixture of the original “West Europe” ancestry with individuals of predominantly “North Europe” ancestry, or a by replacement with individuals that were already of mixed ancestry at the time of arrival in the islands (the latter are not uncommon in Viking Age mainland Europe).” In our revisions, we further included the DATES estimations of the timing of admixture in the modern and historical Faroese samples, which pre-date the timing of settlement in both cases. We highlight these points in the Discussion. And, as with the case of the British population, the closely-related ancestral sources for the Faroese founders were likely not so diverged as to have differences in allele frequencies and long-range haplotypes that would disrupt signals of selection from iHS or XP-EHH.

(3) Time scale: It is certainly possible, and in fact likely, that iHS measures selection older than the settlement of the Faroe Islands. In our manuscript, we calculated iHS in both the Faroese and the closely related British cohort, and we highlight in the main Main Text that the top signals, with the exception of LCT, are shared between the two cohorts, indicative of selection that began prior to the population split (Discussion and Results - Signals of Positive Selection). iHS is a commonly calculated statistic, and it is often calculated in a single population without comparing to others, so we feel it is important to show our result demonstrating these shared selection signals. In our revisions, we now clarify in the Discussion the limitations and time-scale at which the iHS statistic may detect selection. As far as XP-EHH, it is a statistic designed to identify differentiated variants that are fixed or approaching fixation in one population but not others. The time-scale of selection that XP-EHH can detect would therefore be dependent on the populations used for comparison. As XP-EHH has the best power to identify alleles that are fixed or approaching fixation in one population but not others, it is less likely to detect older selection events / incomplete sweeps from the source populations. We highlight this point in the Discussion.

(4) Similarly, for the discussion of LCT, I am not convinced that the haplotypes depicted here are on the right scale to reflect processes happening on the Faroes. Given the admixture/population history, it at the very least should be discussed in the context of whether the 13910 allele frequency on the Faroes is at odds with what would be expected based on the admixture sources.

We agree that more investigation into the LCT allele frequency in the other ancient samples may provide some insight into the selection history, particularly in light of ancient admixture. Please note, we did look at the allele frequency of the LCT allele rs4988235 and stated in the main text that it was present at high frequencies in the historical (250BP) Faroese samples. The frequency of this allele in the imputed historical Faroese samples is 82% while the allele is present at ~74% frequency in modern samples. We originally did not report the exact percentage in the main text because the sample size of the historical samples (11 individuals) is small and coverage of ancient samples is low, leading to potential errors in imputation.

However, given the reviewer’s comment, we have now included the frequencies as well as these caveats in the Discussion. We additionally calculated the LCT allele frequency in other ancient samples, and assuming that we had good proxies for the sources at the time of admixture, we calculated the expected allele frequency in the admixed ancestors of the Faroese founders (Discussion), but again note the limitations in using such a calculation in this context.

(5) I am lacking information to evaluate the procedure for turning the outliers into p-values. Both iHS and XP-EHH are ratio statistics, meaning they might be heavy-tailed if one is not careful, and the central limit theorem may not apply. It would be much easier (and probably sufficient for the points being made here) to reframe this analysis in terms of empirical outliers.

Given that there are disagreements on the best approach to reporting selection scan results from the reviewers, in our revision, we have additionally supplied both the standardized iHS / XP-EHH values in Supplementary Fig. S10 as well as these values transformed to p-values in Main Text Fig. 3. Additionally, both outputs are provided in the publicly available selection scan results files. We provide the method for obtaining p-values in the subsection “Selection scan” from the Methods section - we used a method developed earlier by Fariello et al.

(6) Oldest individual predating gene flow: It seems impossible to make any statements based on a single individual. Why is it implausible that this person (or their parents), e.g., moved to the Faroes within their lifetime and died there?

We agree with the reviewer that this is a plausible explanation, and in our revisions, we have updated the Main Text - Discussion to acknowledge this possibility.

**Recommendations for the authors:**

**Reviewing Editor Comments:**

Please note that there was disagreement among the reviewers regarding the reporting of outliers.

As stated in our response to the public reviews, given the disagreement, we include both the empirical selection statistics as well as the converted p-values in the main text, supplement and selection scan files.

**Reviewer #2 (Recommendations for the authors):**

(1) Figure 2:

Define labels / explain why they differ from 1000k populations / make them consistent throughout the manuscript.

We apologize for the error in labels for Figure 2. These are the same populations used in other figures and analyses. We have fixed this in our revisions so that the labels are consistent with the rest of the manuscript.

(2) Figure S2 label:

"The matrix is rescaled after subsetting the individuals, so although the scales are different, the overall structure remains the same." I do not understand this sentence. The samples are different, the scale is different, the apparent pattern is different - what overall structure is supposed to be the same?

We apologize that the language was not clear in the figure label. The scales between panels A and B are different, because popkin rescales the kinship labels after subsetting so that the minimum kinship is zero. This is necessary when subsetting individuals from an already estimated kinship matrix particularly when subsetting from global populations to a single region. From the popkin documentation: “This rescaling is required when subsetting results in a more recent Most Recent Common Ancestor (MRCA) population compared to the original dataset (for example, if the original data had individuals from across the world but the subset only contains individuals from a single continent)” (https://rdrr.io/cran/popkin/man/rescale_popkin.html).

We also described this in the Methods - Population Genetics - Kinship and runs of homozygosity section: “When calculating the kinship matrix for the Faroese WGS cohort only, we used the rescale_kinship() function, which will change the most recent common ancestor and give different absolute values, but the overall relationship structure in the subpopulation remains the same.”

That is, the relative kinship within the Faroese cohort remains consistent, despite the different scale.

It is difficult to see the kinship of Faroese individuals in the larger plot with all cohorts, which is why we subset and visualize the Faroese cohort alone. We have updated the Fig. S2 label language to make this more clear.

(3) "Iron Age Wet Europe"

We have corrected this typo to “Iron Age West Europe.”

I'm confused if the ancient Faroese were part of the imputation panel: Figure 5 legend implies they are, methods imply they are not.

The ancient samples are not imputed with the modern Faroese and reference samples, but they are the imputed data downloaded from Allentoft et al. and merged with the modern Faroese cohort. We specify that we downloaded imputed ancient samples in both the Methods - Fine-scale structure estimation using ancient genomes and in the Main Text - Results - Fine-Scale Structure and Connections to Ancient Genomes. The description of the imputation panel in the Methods - Bioinformatics - Variant calling and imputation refers only to the modern samples.

(4) Kinship:

The kinship of the Faroes is useful (and nice) as a QC analysis showing the genetic data matches the expectations from the pedigree. I don't know what I should learn from the kinship of the 1000kg samples (I'd assume one could learn something about bottleneck strength from this), but it's not developed/discussed.

The global kinship matrix provides complementary information to PCA and ROH, as another way to quantify and visualize the relationships within and between populations. Additionally, as the reviewer mentioned, bottlenecks increase kinship within populations. Given that popkin estimates kinship measured from a Most Recent Common Ancestor, we can best observe this increase in kinship when comparing to other global populations. We more clearly delineate what can be observed from Fig. S2A versus Fig. S2B in the Results - Population Structure and Relatedness.

Reference

(1) Gretzinger, J. et al. The Anglo-Saxon migration and the formation of the early English gene pool. Nature 610, 112–119 (2022)

(2) Leslie, S. et al. The fine-scale genetic structure of the British population. Nature 519, 309–314 (2015).
